# Comparison of effectiveness and safety of lasmiditan and CGRP-antagonists for the acute treatment of migraine in adults: systematic review and network meta-analysis of randomised trials

**DOI:** 10.1186/s10194-024-01723-4

**Published:** 2024-02-05

**Authors:** Xinxin Deng, Liying Zhou, Cui Liang, Xue Shang, Xu Hui, Wendi Liu, Shanshan Liang, Yongsheng Wang, Meng Xu, Kangle Guo, Kehu Yang, Xiuxia Li

**Affiliations:** 1 Health Technology Assessment Center/Evidence-Based Social Science Research Center, School of Public Health, Lanzhou University, 199 Donggang West Road, Lanzhou, 730000 China; 2 Evidence Based Medicine Center, School of Basic Medical Sciences, Lanzhou University, 199 Donggang West Road, Lanzhou, 730000 China; 3 Key Laboratory of Evidence Based Medicine and Knowledge Translation of Gansu Province, Lanzhou, 730000 China; 4 School of Public Health and Emergency Management, Southern University of Science and Technology, Shenzhen, China; 5 Department of infection management, Gansu Provincial Hospital, Lanzhou, 730000 China

**Keywords:** Acute treatments, Migraine, Lasmiditan, CGRP, Network meta-analyses, Efficacy

## Abstract

**Objective:**

To compare the outcomes associated with the use of lasmiditan, rimegepant, ubrogepant, and zavegepant for the acute management of migraine headaches.

**Methods:**

We searched four electronic databases from database inception to August 31, 2023, to identify randomized controlled trials (RCTs) that report efficacy and safety for the acute treatment of migraine. The risk of bias in the included RCTs was evaluated according to the Cochrane tool, and the certainty of evidence using the CINeMA approach. We conducted frequentist network meta-analyses (NMA) to summarise the evidence. Data were analyzed using R-4.3.1.

**Results:**

A total of 18 eligible studies including 10 different types of interventions with 22,429 migraine patients were included. NMA results showed that compared to ubrogepant (25 mg and 50 mg) and zavegepant, lasmiditan (100 mg and 200 mg) exhibits an elevated probability of achieving pain relief within a 2-hour interval. Similarly, relative to zavegepant, rimegepant (75 mg) and ubrogepant (50 mg and 100 mg) demonstrate an enhanced likelihood of sustaining pain relief over a 24-hour period. Furthermore, in contrast to ubrogepant (25 mg) and lasmiditan (50 mg), rimegepant (75 mg) presents a heightened probability of achieving freedom from photophobia within 2 h. Regarding safety, lasmiditan carries the highest risk of adverse events, which are associated with an increased incidence of adverse effects, including dizziness, somnolence, asthenia, paresthesia, and fatigue.

**Conclusions:**

In this NMA, a spectrum of evidence ranging from very low to high levels underscores the favorable efficacy and tolerability of rimegepant 75 mg and ubrogepant 100 mg, positioning them as potential candidates for the acute management of migraine. Concurrently, lasmiditan (100 mg and 200 mg) exhibits notable efficacy, albeit accompanied by an increased susceptibility to adverse events. These findings should still be approached with caution, primarily due to the intrinsic limitations associated with indirect comparisons.

**Supplementary Information:**

The online version contains supplementary material available at 10.1186/s10194-024-01723-4.

## Introduction

Migraine is a primary headache disorder most often characterized by a unilateral headache, with or without aura [1, 2]. Common associated symptoms include nausea, vomiting, photophobia, phonophobia, blurred vision, and various other physical, mental, and psychological manifestations, which can persist for 4–72 h [3]. Based on prevalence modeling by the Global Burden of Diseases (GBD), it is estimated that approximately 1.04 billion people worldwide experience migraines, with an age-standardized prevalence rate of around 14.4%. The economic impact of migraines is substantial, with an annual cost of almost $17 billion in the United States and an estimated €27 billion per year in Europe, encompassing both direct and indirect expenses, as well as social costs [4, 5]. 

Migraine management comprises both acute and preventive treatments. The goal of acute treatment is to swiftly and effectively alleviate headaches and associated symptoms, restore functional capacity, and minimize the need for rescue medications and the occurrence of adverse events (AEs). Triptans currently stand as the standard of care for addressing acute migraine attacks of moderate to severe intensity [6]. Nevertheless, they are contraindicated for individuals with cardiovascular disease [7]. Moreover, many patients experience inadequate efficacy or tolerance with triptan therapy, creating a substantial unmet need for an alternative acute migraine treatment [8]. This need led to the development of lasmiditan, a potent and selective agonist of the 5-HT1F receptor. Lasmiditan has received approval from the Food and Drug Administration (FDA) and the European Medicines Agency (EMA) for the acute treatment of migraines in adults, both with and without aura [9]. 

Calcitonin gene-related peptide (CGRP) is a neurotransmitter with vasodilatory effects. In the throes of a migraine attack, the release of CGRP significantly surges, emerging as a pivotal trigger for migraines [10]. Consequently, inhibition of the CGRP signaling pathway is a novel mechanism of action for the acute treatment of migraine, with the CGRP receptor now at the forefront of drug development in this area [11, 12]. The FDA has recently approved several innovative treatments for acute migraine. These novel treatments include the CGRP receptor antagonists rimegepant, [13] ubrogepant, [14, 15] and zavegepant [16]. 

Although these medications have demonstrated their effectiveness in treating acute migraine attacks in various placebo-randomised controlled trials (RCTs), [17–23] direct head-to-head RCT comparisons are currently absent. Network meta-analyses (NMA) encompasses a broader spectrum of studies, facilitating indirect comparisons among treatments that have not undergone direct head-to-head evaluation. This approach offers a more comprehensive perspective on the relative efficacy of various treatments, enhancing the statistical power and precision of the results. The use of network diagrams in NMA can provide a visual representation of the network of evidence, which can in turn help identify gaps in the evidence base and inform future research priorities. Additionally, NMA permits the ranking of interventions based on their effectiveness. Therefore, the objective of this study is to conduct a systematic review and NMA to evaluate and compare the therapeutic benefits and safety profiles of 5-HT1F receptor agonists with CGRP antagonists for the treatment of acute migraine attacks.

## Methods

We followed the Preferred Reporting Items for Systematic Reviews and Meta-Analyses (PRISMA) [24] reporting guidelines and had a registered protocol (PROSPERO-ID: CRD42023467187).

### Search strategy

PubMed, Web of Science, Embase, and Cochrane Central Register of Controlled Trials (CENTRAL) were systematically searched for RCTs published from database inception to August 31, 2023. The supplementary search was completed by searching the World Health Organization International Clinical Trials Registry Platform (https://www.genengnews.com/) and Clinical Trials (https://www.clinicaltrials.gov/). The search strategy used the following terms: (migraine OR headache) AND (lasmiditan OR Rimegepant OR ubrogepant OR zavegepant) AND (random* OR blind* OR singleblind* OR doubleblind* OR tripleblind* OR RCT* OR control*). Complete search strategies are listed in the Table S1.

### Study selection

Eligible studies were RCTs that assessed the drugs lasmiditan, rimegepant, ubrogepant, and zavegepant for the acute treatment of migraine in adults, both with and without aura. Exclusion criteria were as follows :1) case reports, letters, comments, conference abstracts and review articles; 2) the full text was not available; 3) articles published in languages other than English. Appendix Table S2 presents the detailed eligibility criteria.

**Fig. 1:**
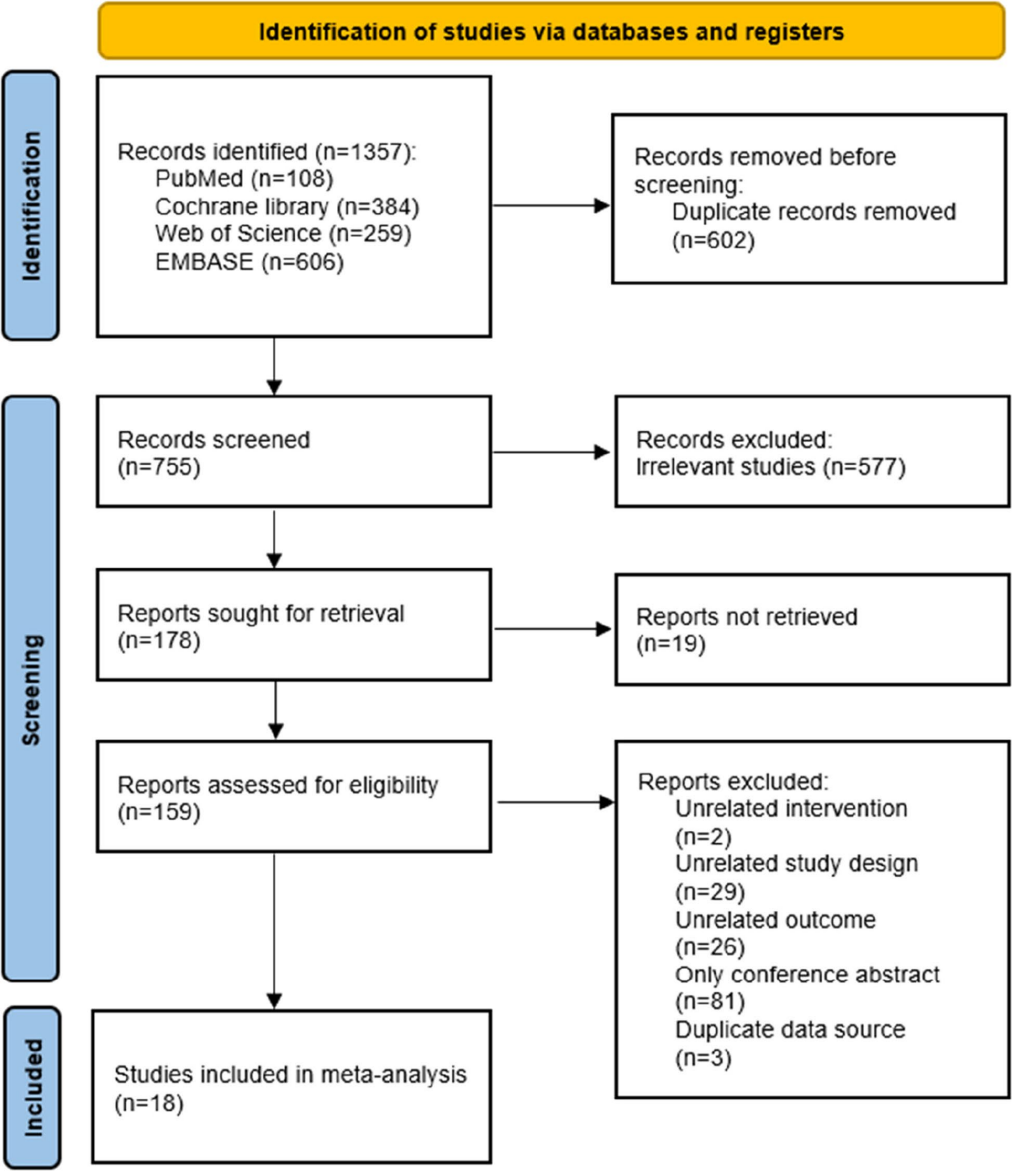
Flow diagram of the literature screening process and results

Two reviewers (XXD and LYZ) independently completed level 1 (title and abstract) and level 2 (full-text) screening for articles using Endnote 20 (Thomson Corporation; Stanford, CT, USA) literature management software. A pilot exercise was conducted initially for both levels of screening to ensure consistency between reviewers. Discrepancies were settled by discussionwith a third reviewer (XXL). We recorded the selection process in suffcient detail to complete a PRISMA flow diagram (Fig. 1).

### Data extraction

Two reviewers independently extracted the following data from the eligible studies by using a pre-specified data form: general information (name of first author, year of publication, and country/region), participants (sample size, gender, age, and diagnostic criteria for migraine), comparator details (medication name, dosage, and follow-up time), outcome data (efficacy outcomes data and safety outcomes), and study results (relative risks (RRs), if available).

### Risk of bias

Two reviewers (XXD and CL) independently and in duplicate assessed the risk of bias for each RCT using the version 2 Cochrane risk-of-bias tool (ROB2) [25]. Any disagreement in the rating was resolved by discussion or with a third reviewer (XXL). The ROB2 assessment tool comprises domains for evaluating bias arising from the randomization process, bias due to deviations from intended interventions, bias due to missing outcome data, bias in the measurement of the outcome, and bias in the selection of the reported result. These domains were judged using high, some concerns, or low risk of bias judgments. Judging a result to be at a particular level of risk of bias for an individual domain implies that the result has an overall risk of bias at least this severe.

### Data analysis

We conducted a random-effects model NMA with a frequentist framework [26] using the netmeta statistical package in R version 4.3.1 (R Core Team, Vienna, Austria). In addition, we drew network plots in Stata version 15.1 (StataCorp, College Station, TX) to visually represent all interventions [27]. In these plots, the size of nodes represents the number of individuals and the thickness of lines between nodes represents the number of studies. The outcomes were dichotomous data, and we pooled them as RR and 95% CI in a random-effect model. We performed an overall inconsistency test and used the P-value to determine the consistency level [28, 29]. We used the node splitting method to generate effect sizes and credible intervals for the indirect comparison and to conduct a statistical test for incoherence (also known as inconsistency) between direct and indirect estimates [30, 31]. If *p* < 0.05, local inconsistency was considered to exist. Important inconsistencies can threaten the validity of the results; if present, the possible sources of disagreement were explored and identified. We used the surface under the cumulative ranking curve (SUCRA) to rank the intervention hierarchy in the NMA. The SUCRA value closer to 1 indicates a higher probability of a treatment being among the top-ranked treatments or the best option overall.

### Certainty assessment

We used a semiautomated web application (http://cinema.ispm.ch), Confidence in Network Meta-analysis (CINeMA), [32] for the certainty of evidence for each outcome. The tool considered the following six areas: intra-study bias (bias risk), inter-study bias (publication bias or report bias), indirectness, imprecisions, heterogeneity, and inconsistency. Each area was rated as no concern (no downgrade), some concern (one level downgrade), and major concern (two levels downgrade) based on the severity of the bias. Finally, the evidence for each pair of comparisons was determined as high, moderate, low, or very low according to the degree of degradation [29, 33]. 

## Results

### Search results and study characteristics

Our electronic search yielded 1357 unique records, of which 159 were potentially eligible (Fig. 1). After full text reviews, we excluded 141 (Appendix Table S3 for exclusion reasons): two were unrelated interventions, 29 were unrelated study designs, 26 were unrelated outcomes, 81 were only conference abstract, and two were duplicated data sources. Finally, 2 open-label and 16 double-blinded RCTs comparing 10 interventions with each other or with placebo were included for analysis. Among them, 5 were phase 2 trials,13 were phase 3 trials. The included RCTs were conducted across different countries like Japan, China, USA, Finland, and the United Kingdom from 2014 to 2023 (all 18 of the included studies were conducted at multiple centers). The studies included a total of 22,429 participants (range: 322 to 2,583). Across all studies, females represented about 79% of the included patients, and the mean age ranged from 36.0 to 45.7 years. All study participants fulfilled the International Headache Society (IHS) or International Classification of Headache Disorders (ICHD) diagnostic criteria (at least a 1-year history of migraine with or without aura). Appendix Table S4 summarises the characteristics of the included study.

### Risk of bias

During the literature bias assessment, 13 studies were assessed as low bias risk with the RoB 2.0 tool due to their high quality. However, three studies were assessed as high bias risk since we have concerns about the randomization process, deviations from intended interventions, and measurement of the outcome of Brandes 2020, Brandes 2019, and Lipton 2022. Two studies were found to have a moderate risk of bias. The risk of bias graph is shown in Appendix Fig. S1.

### Inconsistency analysis

The overall and local inconsistency tests were conducted to assess consistency. Most fitted models converged well, except for the placebo versus ubrogepant 50 mg and ubrogepant 100 mg comparisons in achieving freedom from phonophobia at 2 h, which showed a statistically significant difference (*p* < 0.05) (Appendix Table S5).

### Efficacy analysis

Among the 18 RCTs that assessed migraine, the seven most common outcome measures were pain freedom at 2 h, pain relief at 2 h, most bothersome symptom (MBS) freedom at 2 h, sustained pain freedom over 24 h, sustained pain relief over 24 h, freedom from photophobia at 2 h, and freedom from phonophobia at 2 h. Hence, they were identified as the outcome index for NMA in the present study.

### Pain freedom at 2 h

A total of 16 RCTs and nine treatment nodes were included for the outcome of pain freedom at 2 h (Appendix Fig. S2). Lasmiditan was the most commonly investigated intervention (8 RCTs). Compared with placebo, lasmiditan 100 mg, 200 mg (RR, 1.54 [95% CI, 1.21–1.99]; RR, 1.85 [95% CI, 1.46–2.40]), rimegepant 75 mg (RR, 1.82 [95% CI, 1.30–2.55]), and ubrogepant 25 mg, 50 mg, 100 mg (RR, 1.64 [95% CI, 1.06–2.58]; RR, 1.64 [95% CI, 1.18–2.46]; RR, 1.96 [95% CI, 1.31–2.97]) demonstrated statistically significant higher odds of including pain freedom at 2 h, and lasmiditan 200 mg (RR, 1.43 [95% CI, 1.02–2.03]) presented higher odds of inducing pain freedom at 2 h than lasmiditan 50 mg. The results of NMA are shown in Table 1. Ubrogepant 100 mg had the highest SUCRA value for pain freedom at 2 h at 0.79, followed by lasmiditan 200 mg (SUCRA, 0.74), and rimegepant 75 mg (SUCRA, 0.69) (Appendix Table S6).

**Table 1:**
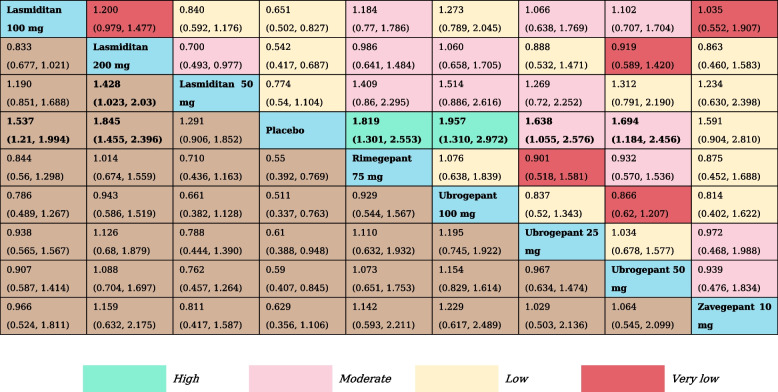
The results of network meta-analysis for pain freedom at 2  h

All effect sizes were presented using risk ratios (RRs) values and 95% confidence intervals. In each column, each effect size was the result of that intervention compared to any other intervention. Cells in bold print indicate significant results

### Pain relief at 2 h

We analyzed pain relief at the 2 h by reviewing 12 RCTs and assessing 11 treatment nodes (Appendix Fig. S3). Compared with placebo, lasmiditan 100 mg (RR, 1.44 [95% CI, 1.34–1.55]), lasmiditan 200 mg (RR, 1.43 [95% CI, 1.33–1.54]), lasmiditan 50 mg (RR, 1.28 [95% CI, 1.17–1.40]), rimegepant 75 mg (RR, 1.37 [95% CI, 1.28–1.46]), ubrogepant 100 mg (RR, 1.27 [95% CI, 1.12–1.43]) ubrogepant 50 mg (RR, 1.27 [95% CI, 1.16–1.39]), ubrogepant 25 mg (RR, 1.23 [95% CI, 1.09–1.39]), zavegepant 10 mg (RR, 1.16 [95% CI, 1.06–1.24]) and zavegepant 20 mg (RR, 1.16 [95% CI, 1.03–1.30]) showed statistically significant higher odds of inducing a reduction in headache pain at 2 h. Zavegepant 5 mg was not significantly different from placebo. Otherwise, lasmiditan 100 mg and lasmiditan 200 mg were associated with statistically significant higher odds of achieving a reduction in headache pain at 2 h versus ubrogepant 25 mg, ubrogepant 50 mg, and all doses of zavegepant (Table 2). SUCRA rankings indicate that Lasmiditan at doses of 100 mg (SUCRA, 0.94) and 200 mg (SUCRA, 0.91) offer the highest likelihood of effectively relieving pain within 2 h (Appendix Table S6).

**Table 2:**
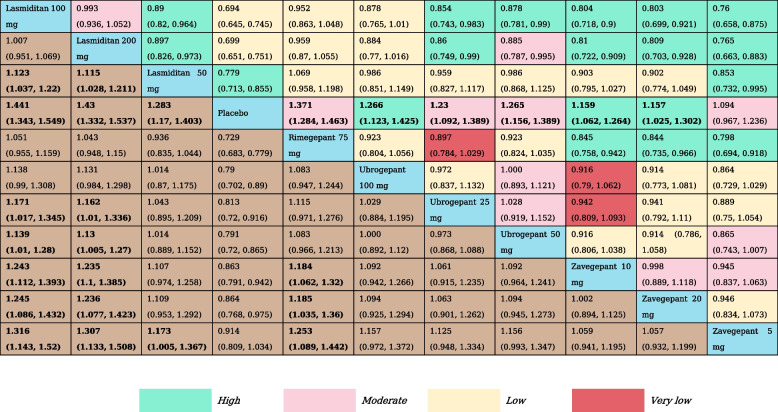
The results of network meta-analysis for pain relief at 2  h

All effect sizes were presented using RRs values and 95% confidence intervals. In each column, each effect size was the result of that intervention compared to any other intervention. Cells in bold print indicate significant results

### MBS freedom at 2 h

For this specific outcome, we included a total of 10 RCTs and nine treatment nodes (Appendix Fig. S4). Compared with placebo, lasmiditan 100 mg (RR, 1.17 [95% CI, 1.00-1.39]), lasmiditan 200 mg (RR, 1.20 [95% CI, 1.03–1.43]), rimegepant 75 mg (RR, 1.40 [95% CI, 1.17–1.68]), ubrogepant 100 mg (RR, 1.37 [95% CI, 1.01–1.82]) ubrogepant 50 mg (RR, 1.40 [95% CI, 1.11–1.78]) demonstrated statistically significant higher odds of inducing MBS freedom at 2 h (Table 3). SUCRA values confirmed rimegepant 75 mg and ubrogepant 50 mg to be the best treatment, with a SUCRA value of 0.79, followed by ubrogepant 100 mg (SUCRA, 0.71) (Appendix Table S6).

**Table 3:**
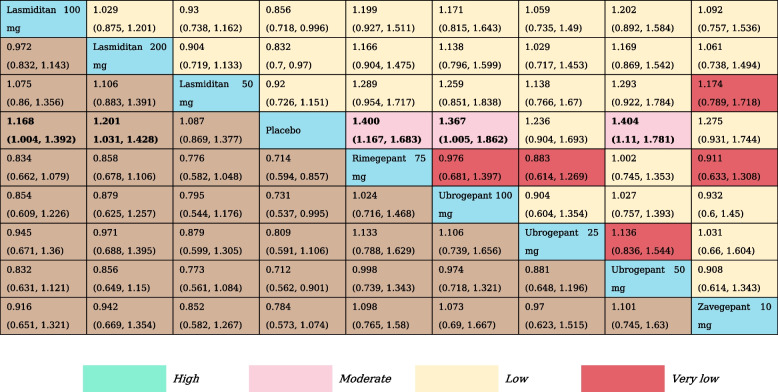
The results of network meta-analysis for MBS freedom at 2  h

All effect sizes were presented using RRs values and 95% confidence intervals. In each column, each effect size was the result of that intervention compared to any other intervention. Cells in bold print indicate significant results

### Sustained pain freedom over 24 h

In total, 13 RCTs and 11 treatment nodes were included for this outcome (Appendix Fig. S5). The pooled network outcome that was obtained by comparing each intervention against the placebo revealed that all interventions were statistically equivalent to the placebo (Table 4). Nonetheless, the three top-ranked interventions for sustained pain freedom over 24 h were Ubrogepant 100 mg (SUCRA, 0.74), followed by Lasmiditan 200 mg (SUCRA, 0.65), and Ubrogepant 50 mg (SUCRA, 0.59) (Appendix Table S6).

**Table 4:**
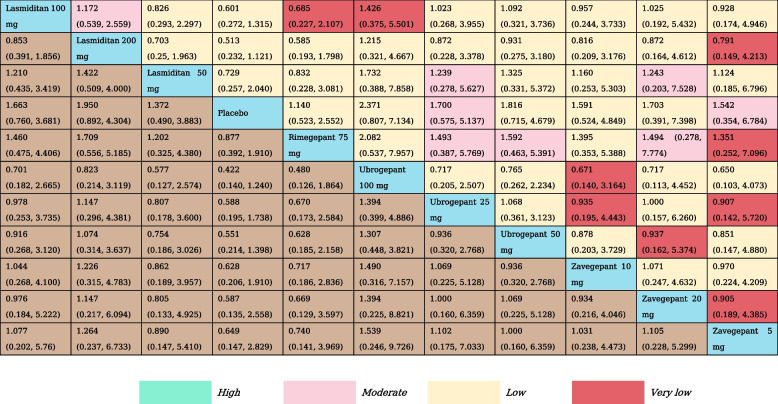
The results of network meta-analysis for sustained pain freedom over 24  h

All effect sizes were presented using RRs values and 95% confidence intervals. In each column, each effect size was the result of that intervention compared to any other intervention. Cells in bold print indicate significant results

### Sustained pain relief over 24 h

For the evaluation of sustained pain relief over 24 h, we examined data from eight RCTs and eight treatment nodes. (Appendix Fig. S6). Compared with placebo, all gepants including rimegepant 75 mg (RR, 1.66 [95% CI, 1.48–1.88]) and any dose of ubrogepant (ubrogepant 25 mg: RR, 1.52 [95% CI, 1.25–1.83]; ubrogepant 50 mg: RR, 1.72 [95% CI, 1.47–2.01]; ubrogepant 100 mg: RR, 1.78 [95% CI, 1.45–2.13]) and zavegepant (zavegepant 5 mg: RR, 1.24 [95% CI, 1.02–1.50]; zavegepant 10 mg: RR, 1.21 [95% CI, 1.06–1.40]; zavegepant 20 mg: RR, 1.26 [95% CI, 1.04–1.53]) were associated with statistically significant higher odds of achieving sustained pain relief over 24 h versus placebo (Table 5). Zavegepant 20 mg had the highest SUCRA value for the outcome of sustained pain relief over 24 h at 1.00, followed by ubrogepant 100 mg (SUCRA, 0.76), and ubrogepant 50 mg (SUCRA, 0.70) (Appendix Table S6).

**Table 5:**
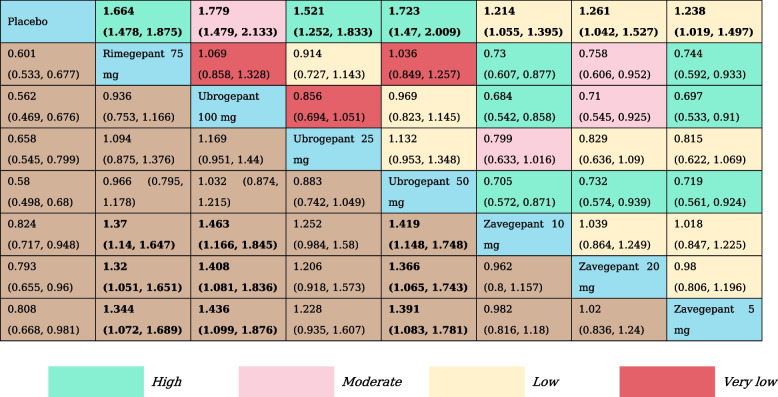
The results of network meta-analysis for sustained pain relief over 24  h

All effect sizes were presented using RRs values and 95% confidence intervals. In each column, each effect size was the result of that intervention compared to any other intervention. Cells in bold print indicate significant results

### Freedom from photophobia at 2 h

We incorporated 11 RCTs and 11 treatment nodes into the analysis for this outcome(Appendix Fig. S7). Compared with placebo, lasmiditan 100 mg (RR, 1.27 [95% CI, 1.12–1.44]), lasmiditan 200 mg (RR, 1.30 [95% CI, 1.15–1.48]), rimegepant 75 mg (RR, 1.53 [95% CI, 1.31–1.79]), ubrogepant 100 mg (RR, 1.34 [95% CI, 1.17–1.56]) ubrogepant 50 mg (RR, 1.30 [95% CI, 1.41–1.51]), zavegepant 10 mg (RR, 1.25 [95% CI, 1.05–1.47]) and zavegepant 20 mg (RR, 1.29 [95% CI, 1.02–1.62]) showed statistically significant higher odds of inducing freedom from photophobia at 2 h. Rimegepant 75 mg (RR, 1.31 [95% CI, 1.06–1.65]; RR, 1.42 [95% CI, 1.02–2.06]) was associated with higher odds of achieving Freedom from photophobia at 2 h compared with lasmiditan 50 mg and ubrogepant 25 mg (Table 6). The three top-ranked interventions for achieving freedom from photophobia at 2 h included rimegepant 75 mg (SUCRA, 0.96), ubrogepant 100 mg (SUCRA, 0.72), and lasmiditan 200 mg (SUCRA, 0.64) (Appendix Table S6).

**Table 6:**
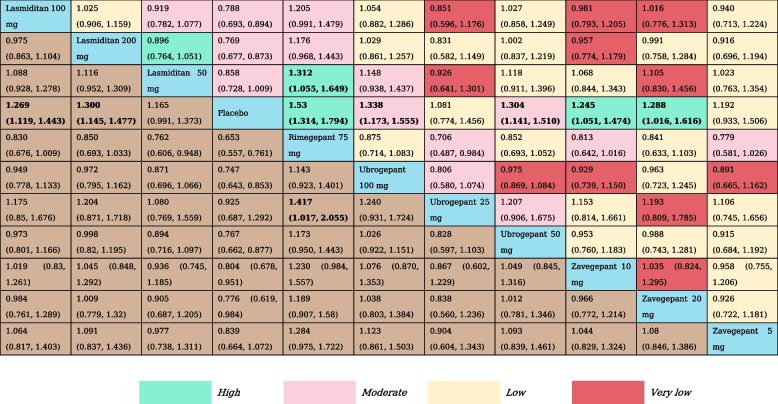
The results of network meta-analysis for freedom from photophobia at 2  h

All effect sizes were presented using RRs values and 95% confidence intervals. In each column, each effect size was the result of that intervention compared to any other intervention. Cells in bold print indicate significant results

### Freedom from phonophobia at 2 h

A total of 11 RCTs and 11 treatment nodes were considered in assessing freedom from phonophobia at 2 h (Appendix Fig. S8). Compared with placebo, rimegepant 75 mg (RR, 1.51 [95% CI, 1.20–1.90]) and ubrogepant 50 mg (RR, 1.30 [95% CI, 1.07–1.60]) showed statistically significant higher odds of inducing freedom from phonophobia at 2 h (Table 7). According to SUCRA, rimegepant 75 mg (SUCRA, 0.90) was associated with the highest probability of effectiveness on freedom from photophobia at 2 h, followed by ubrogepant 50 mg (SUCRA, 0.69), and zavegepant 10 mg (SUCRA, 0.64) (Appendix Table S6).

**Table 7:**
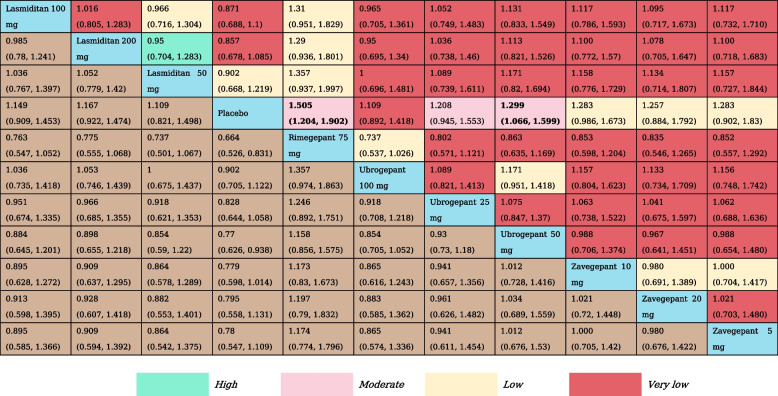
The results of network meta-analysis for freedom from phonophobia at 2  h

All effect sizes were presented using RRs values and 95% confidence intervals. In each column, each effect size was the result of that intervention compared to any other intervention. Cells in bold print indicate significant results

### Safety outcomes

**Fig. 2:**
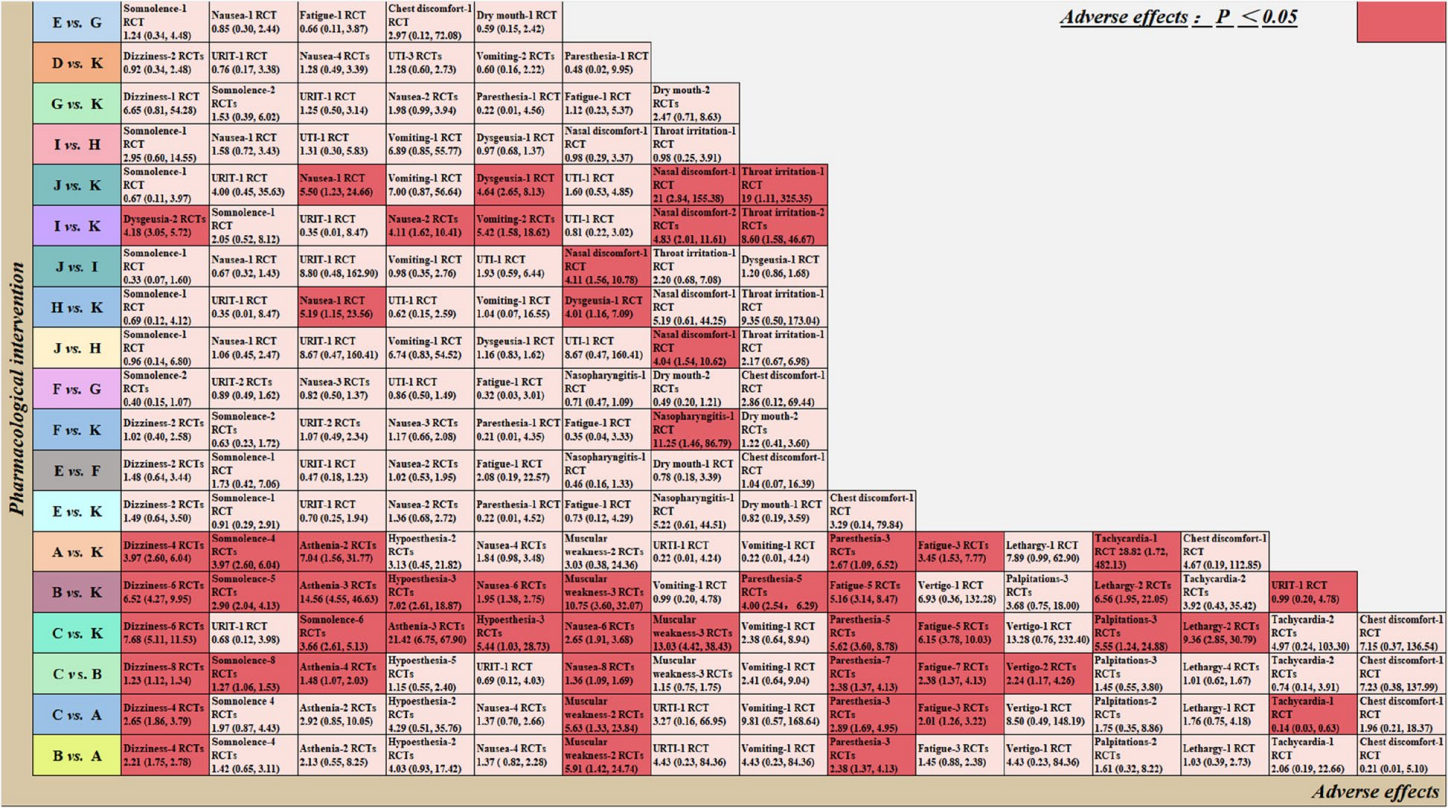
Major adverse events included in the study report. URTI: upper respiratory tract infection; UTI: urinary tract infection; A: lasmiditan 50 mg; B: lasmiditan 100 mg; C: lasmiditan 200 mg; D: rimegepant 75 mg; E: ubrogepant 25 mg; F: ubrogepant 50 mg; G: ubrogepant 100 mg; H: zavegepant 5 mg; I: zavegepant 10 mg; J: zavegepant 20 mg; K: placebo

In terms of safety analysis, we incorporated data from 18 studies encompassing 19 different AEs. During the treatment period, the occurrence of any adverse effect was significantly more likely with lasmiditan. Regarding specific AEs, lasmiditan had increased risks for dizziness, somnolence, asthenia, and fatigue compared with placebo. Lasmiditan 200 mg demonstrated heightened risks of dizziness (RR, 2.65 [95% CI, 1.86–3.79]; RR, 1.23 [95% CI, 1.12–1.34]), paresthesia (RR, 2.89 [95% CI, 1.69–4.95]; RR, 2.38 [95% CI, 1.37–4.13]), and fatigue (RR, 2.01 [95% CI, 1.26–3.22]; RR, 2.38 [95% CI, 1.37–4.13]) compared to lasmiditan 50 mg and lasmiditan 100 mg. In the comparison of rimegepant 75 mg versus placebo, no statistically significant adverse reactions were observed. There was also no significant difference between any dose of ubrogepant in terms of adverse reactions, but the risk of nasopharyngitis was higher for ubrogepant 50 mg (RR, 11.25 [95% CI, 1.46, 86.79]) compared with placebo. Based on the effect size, we observed more nausea and dysgeusia related to any dose of zavegepant compared with placebo. Zavegepant 10 mg exhibited no statistically significant adverse reactions when compared to zavegepant 5 mg. Notably, zavegepant 20 mg had increased risks for nasal discomfort (RR, 4.11 [95% CI, 1.56–10.78]; RR, 4.04 [95% CI, 1.54–10.62]) when compared to zavegepant 10 mg and zavegepant 5 mg (Fig. 2).

### Certainty of evidence

The certainty of evidence for each outcome is detailed in Appendix Table S7. In our analysis of all seven outcomes, we observed that the confidence in the evidence for 49% of comparisons with placebo was rated as either low or very low (as demonstrated in Appendix Fig. S9). This was the case for 70% of instances when making pairwise comparisons between two drugs, primarily attributable to factors such as within-study bias, imprecision, heterogeneity, or incoherence.

## Discussion

### Summary of the main results

In this systematic review and NMA, we conducted an assessment of the efficacy and safety of four medications approved by the U.S. FDA for the acute treatment of adult migraines, including the 5-HT1F receptor agonist (lasmiditan) and the CGRP antagonists (rimegepant, ubrogepant, and zavegepant). Additionally, we determined their efficacy rankings using the SUCRA curve. Our analysis drew from data originating in 18 RCTs encompassing seven distinct treatment outcomes. The study’s findings indicate that relative to a placebo, zavegepant (10 mg and 20 mg) exhibited an elevated RR for pain relief at 2 h, sustained pain relief over 24 h, and freedom from photophobia at 2 h. Lasmiditan (100 mg and 200 mg), rimegepant 75 mg, and ubrogepant (50 mg and 100 mg) demonstrated notable efficacy in terms of pain relief, MBS freedom, freedom from photophobia at 2 h, and sustained pain relief over 24 h. In the SUCRA rankings, rimegepant 75 mg was established as the preferred intervention for three of the treatment outcomes. Furthermore, no significant disparities in AE occurrences were noted between rimegepant 75 mg and the placebo. These findings provide strong indications that rimegepant 75 mg may constitute a current, effective, and safe option for the management of acute migraine in adults.

### Comparison with other studies

Regarding these research findings, our results align with previous systematic reviews and meta-analyses, indicating that, in comparison to a placebo, lasmiditan, rimegepant, and ubrogepant have shown significant benefits in alleviating and relieving headache within 2 h [34–36]. However, our study goes beyond the existing literature by conducting a more comprehensive analysis of these drugs’ effects and introducing a new medication, zavegapant, which received FDA approval for acute treatment of adult migraine in March 2023 [16]. Specifically, we employed an NMA approach to quantitatively compare the effects of different intervention dosages, considering both direct and indirect evidence. This approach allowed us to identify the most effective intervention type and the influence of different interventions on various outcomes. Moderate certainty evidence showed that in comparison to lasmiditan 50 mg, lasmiditan 200 mg demonstrated an elevated likelihood of achieving pain freedom within 2 h, with no significant differences observed in pairwise comparisons among the other treatments. Regarding pain relief within 2 h, moderate to high certainty evidence showed that lasmiditan (100 mg and 200 mg) exhibited a higher RR compared to ubrogepant (25 mg and 50 mg). Furthermore, high certainty evidence suggested that lasmiditan (100 mg and 200 mg) and rimegepant 75 mg showed higher RR when compared to any dosage of zavegapant. Conversely, a separate study indicated that lasmiditan 100 mg and 200 mg had a higher probability of achieving pain freedom and pain relief within 2 h when compared to rimegepant 75 mg [37]. It is worth noting that, based on a study by Puledda and colleagues, their NMA of three Phase III randomsized controlled trials assessed the effectiveness of three novel drugs (lasmiditan, rimegepant, and ubrogepant) for the acute treatment of migraine attacks, finding that lasmiditan, particularly at higher doses (100 and 200 mg), outperformed all other treatments in achieving pain freedom within 2 h [38]. This discrepancy may be attributed to the fact that the study by Puledda et al. included only three Phase III trials, whereas our study encompassed a greater number of trials with a larger sample size. Nevertheless, it is important to emphasize that there is currently no direct comparative data between these drugs. Therefore, while NMA offers valuable indirect comparisons, it cannot replace direct comparisons in clinical research. Hence, further direct comparative studies will be needed for a more in-depth exploration of this issue.

Regarding MBS freedom at 2 h, our study findings align with those of study, as they both support the notion that, when compared to a placebo, lasmiditan (100 mg and 200 mg), ubrogepant (50 mg and 100 mg), and rimegepant 75 mg demonstrate higher RR values [38]. Notably, Polavieja et al. found that lasmiditan 200 mg exhibited a numerically higher efficacy than both rimegepant and ubrogepant for achieving freedom from MBS [37]. Otherwise, there was very low to moderate-level evidence that no intervention showed a clear advantage in terms of sustained pain freedom over 24 h. According to the SUCRA values, ubrogepant 100 mg emerges as the most likely candidate for the best intervention in achieving 24-hour sustained pain disappearance. In contrast, Puledda et al. found that rimegepant and lasmiditan 200 mg outperformed other interventions in terms of sustained pain freedom at 24 h [38]. Polavieja et al. on the other hand, observed that lasmiditan 200 mg numerically surpassed rimegepant and ubrogepant but did not reach statistical significance [37]. Additionally, Gao et al. included four RCTs to evaluate the efficacy of rimegepant in the acute treatment of migraine. They found that after 24 h of rimegepant administration, patients experienced a 9.8% increase in sustained pain freedom compared to the placebo [34]. Furthermore, Johnston et al. conducted a comparative analysis of the safety and effectiveness of rimegepant, ubrogepant, and lasmiditan for the treatment of acute migraine. Their findings indicated that, in terms of pain-free intervals ranging from 2 to 24 h, rimegepant outperformed lower doses of lasmiditan and ubrogepant [39]. Gao et al. found that after 24 h of rimegepant administration, patients experienced a remarkable 17.8% increase in sustained pain relief compared to the placebo [34]. In the domain of sustained pain relief over 24 h, low to high certainty evidence underscores that, in contrast to a placebo, all interventions yield elevated RR values. This aligns harmoniously with the observations of Johnston’study, which distinctly posits that, within the temporal window of 2 to 24 h post-administration, all active control medications surpass the placebo [39]. However, in comparison to zavegepant, rimegepant 75 mg and ubrogepant (50 mg and 100 mg) manifest conspicuous advantages in maintaining pain relief over the 24-h horizon.

In our NMA, moderate certainty evidence suggested that lasmiditan 200 mg demonstrated superiority in freedom from photophobia compared to placebo. However, moderate to high certainty evidence showed that a comparable effect was almost equally achieved not only with lasmiditan 100 mg but also with rimegepant, ubrogepant (50 mg and 100 mg), and zavegepant (10 mg and 20 mg). In the context of freedom from photophobia 2 h after medication administration, the results of Puledda et al. were consistent with our findings [38]. Furthermore, support for our results was derived from a study by Zhang et al., which specifically indicated that participants who had taken ubrogepant exhibited a significantly lower prevalence of photophobia than those in the placebo group at the two-hour mark [36]. In addition, a study by Johnston et al. reported that lasmiditan was notably more effective than rimegepant in addressing photophobia at the two-hour point [39]. However, it is worth noting that our study did not observe this level of statistical significance. In the freedom from phonophobia outcome, rimegepant was equal to ubrogepant 50 mg, whereas lasmiditan was not as effective. Similarly, in line with our findings, the study by Zhang et al. reported a significantly higher percentage of participants experiencing phonophobia in the ubrogepant group compared to the placebo group 2 h after medication administration [36]. Our study found low and high certainty evidence that zavegepant (10 mg and 20 mg) demonstrated higher RRs compared with placebo for pain relief at 2 h, sustained pain relief over 24 h, and relief from photophobia at 2 h. However, it did not demonstrate a significant therapeutic advantage over other comparator medications across all measured outcomes.

The results from the side effect analysis matched with previous findings [35, 38, 40]. Lasmiditan exhibited the highest risk of AEs among all treatments, notably involving adverse events such as dizziness, somnolence, asthenia, paresthesia and fatigue. Furthermore, significant differences in adverse events were observed among different doses of lasmiditan, with higher doses associated with increased risk of adverse reactions. Additionally, the use of zavegepant was primarily associated with a significant increase in AEs related to dysgeusia, nausea, nasal discomfort, and throat irritation. However, no serious AEs were reported. Our study findings indicate that rimegepant is a relatively safe option for migraine treatment. There was no significant difference observed between rimegepant 75 mg and the placebo group. This observation is supported by a specific study, demonstrating the similarity of rimegepant 75 mg to the placebo group regarding AEs such as nausea, urinary tract infections, and dizziness during migraine treatment [34]. Furthermore, Puledda et al. noted that both Rimegepant 75 mg and Ubrogepant 50 mg formulations exhibited excellent efficacy-to-adverse-effect ratios [38]. Further investigations, including the study by Zhang et al., revealed a similar rate of common AEs between ubrogepant and the placebo group 2 h after administration [36]. Additionally, a meta-analysis regarding the use of calcitonin gene-related peptide receptor antagonists in the acute treatment of migraine demonstrated that ubrogepant was associated with the lowest risk of AEs, with both ubrogepant and rimegepant exhibiting lower toxicity compared to triptans [41]. Although our study reported only one instance where ubrogepant 50 mg led to a higher incidence of Nasopharyngitis compared to the placebo, the safety profile of ubrogepant still requires further confirmation through additional clinical research due to the limitations of the included studies.

### Clinical implications

Our research findings demonstrate that when compared to ubrogepant, zavegepant, and placebo, lasmiditan (100 mg and 200 mg) exhibited superior pain relief efficacy within 2 h. Consequently, for patients seeking rapid relief from pain, considering the utilization of lasmiditan may be deemed a viable option. However, given the elevated risk of AEs associated with lasmiditan, clinicians should diligently monitor patients for adverse reactions post-medication administration and adjust the dosage or consider alternative treatments when necessary. In this regard, rimegepant emerges as a potentially appropriate alternative. For patients aiming to achieve pain freedom within a short timeframe, prioritizing ubrogepant is recommended. Despite ubrogepant 100 mg being identified as the optimal intervention for achieving pain freedom within 2 h, our study results indicate that even ubrogepant 25 mg exhibits efficacy in reaching pain freedom at 2 h. Considering the apparent dose-related central nervous system AEs, we suggest a careful balance between the efficacy and AE risks when considering the clinical application of ubrogepant. For achieving MBS freedom within 2 h and sustained pain relief over 24 h, we advocate for the utilization of rimegepant 75 mg. In both efficacy and safety aspects, rimegepant demonstrates advantages over lower doses of comparators and, in terms of safety, over higher doses of comparators. Additionally, the selection of medication should involve an assessment of the patient’s specific migraine symptoms, allowing for personalized treatment based on the unique characteristics of each patient. For instance, for migraine patients experiencing photophobia and phonophobia, our preference leans towards rimegepant 75 mg. It’s worth noting that for patients facing difficulty swallowing, zavegepant appears to be a promising new candidate. Its commendable performance in pain relief within 2 h, sustained pain relief over 24 h, and relief from photophobia within 2 h is underscored. Furthermore, the intranasal spray formulation for migraine treatment may contribute to an enhanced overall quality of life for migraine patients by reducing the necessity for conventional oral tablets or pills [42]. However, given the limited number of randomized controlled trials conducted on this medication, there is an urgent need for additional clinical trials to comprehensively assess the safety and efficacy of zavegepant and alternative treatment modalities in the acute management of migraines.

### Assessment of evidence quality

There are several findings worth noting about the quality of the evidence. Three of the 18 RCTs included in this NMA were rated as high risk of bias due to the low methodological quality, reducing the overall evidence level. In addition, many comparisons provided low certainty evidence, primarily because of inconsistency and imprecision, but also because of the risk of bias. Potential heterogeneity among the included trials may account for the observed inconsistency. In the case of mixed comparisons, inconsistency tests were conducted to assess the agreement between direct and indirect comparisons. Node-splitting analysis detected local inconsistency, indicating differences between ubrogepant (50 mg and 100 mg) and placebo in terms of achieving freedom from phonophobia at 2 h. This inconsistency in comparisons suggests weak transitivity in indirect comparisons, which may impact the overall effect and ultimately lower the level of evidence. In such situations, it is recommended to rely on effect measures based on direct comparisons. Specifically, statistical heterogeneity may be related to population characteristics and treatment regimens, including differences in sample size, gender, treatment duration, and other factors [43, 44].

### Strengths and limitations

Strengths of this review include our first inclusion of four new drugs for the acute treatment of migraine and the use of NMA to produce reliable estimates of symptoms, such as pain relief and pain freedom in migraineurs using direct and indirect comparisons. We used explicit eligibility criteria; conducted a comprehensive literature search developed with an experienced librarian; performed duplicate assessment of study eligibility, risk of bias, and data extraction; applied the CINeMA approach to rate certainty of evidence; and presented tables of results highlighting certainty of evidence.

Our review has some limitations. The paucity of direct comparisons between different drugs contributed to the low certainty evidence: 10 intervention programmes included 320 paired comparisons across seven outcomes, of which only 115 made direct comparisons, and, of these, only 69 included more than one study. Furthermore, differences in the study designs pertaining to the use of migraine medications, coupled with specific factors including patient age, gender, and baseline health status, may exert an influence on the placebo effect, contributing to a degree of introduced heterogeneity. Consequently, when interpreting the results of NMA, careful consideration of their potential impact remains imperative. Finally, the results of this study are only based on the current published literature, the results may change with the emergence of new research. We will continue to follow up and update within two years.

## Conclusion

In this NMA, a spectrum of evidence ranging from very low to high underscores the rimegepant 75 mg and ubrogepant 100 mg present good efficacy and a favorable tolerability profile, positioning them as prospective choices for the acute management of migraine. Simultaneously, Lasmiditan (100 mg and 200 mg) demonstrates noteworthy efficacy, albeit accompanied by an elevated susceptibility to AEs. These results warrant circumspect evaluation, primarily attributable to the intrinsic constraints inherent in indirect comparative analyses. It is crucial for future research to encompass larger, well-designed RCTs to validate our findings and ascertain the optimal dosage and long-term safety of these medications for patients receiving migraine treatment.

### Supplementary Information

**Additional file 1: Table S1. **Search strategy. **Table S2.** Detailed eligibility criteria for abstract and title screening. **Table S3.** List of excluded studies during full-text eligibility assessment. **Table S4.** Basic characteristics of included studies. **Table S5.** Node splitting test for inconsistency. **Table S6.** SUCRA values for all outcomes. **Table S7.** CINeMA ratings for all comparisons of the seven outcomes. **Fig. S1.** Risk of bias assessment for the included studies. **Fig. S2.** Network plot for pain freedom at 2 h. **Fig. S3.** Network plot for pain relief at 2 h. **Fig. S4.** Network plot for MBS freedom at 2 h. **Fig. S5.** Network plot for sustained pain freedom over 24 h. **Fig. S6.** Network plot for sustained pain relief over 24 h. **Fig. S7.** Network plot for freedom from photophobia at 2 h. **Fig. S8.** Network plot for freedom from phonophobia at 2 h. **Fig. S9.** Confidence in evidence for all drugs compared to placebo. 

## Data Availability

No datasets were generated or analysed during the current study.
