# Supplementary material for: Comparison of effectiveness and safety of lasmiditan and CGRP-antagonists for the acute treatment of migraine in adults: systematic review and network meta-analysis of randomised trials
Source: J Headache Pain. 2024 Feb 5;25(1):16. doi: 10.1186/s10194-024-01723-4 (PMC10840250; doi:10.1186/s10194-024-01723-4)
Supplement: Supplementary file 1 — Additional file 1: Table S1. Search strategy. Table S2. Detailed eligibility criteria for abstract and title screening. Table S3. List of excluded studies during full-text eligibility assessment. Table S4. Basic characteristics of included studies. Table S5. Node splitting test for inconsistency. Table S6. SUCRA values for all outcomes. Table S7. CINeMA ratings for all comparisons of the seven outcomes. Fig. S1. Risk of bias assessment for the included studies. Fig. S2. Network plot for pain freedom at 2 h. Fig. S3. Network plot for pain relief at 2 h. Fig. S4. Network plot for MBS freedom at 2 h. Fig. S5. Network plot for sustained pain freedom over 24 h. Fig. S6. Network plot for sustained pain relief over 24 h. Fig. S7. Network plot for freedom from photophobia at 2 h. Fig. S8. Network plot for freedom from phonophobia at 2 h. Fig. S9. Confidence in evidence for all drugs compared to placebo. [file 10194_2024_1723_MOESM1_ESM.docx]

**Supplementary Appendix**

Content

[Table S1: Search strategy 3](#_Toc155599463)

[Table S2: Detailed eligibility criteria for abstract and title screening 6](#_Toc155599466)

[Table S3: List of excluded studies during full-text eligibility assessment 7](#_Toc155599467)

[Table S4: Basic characteristics of included studies 15](#_Toc155599468)

[Table S5: Node splitting test for inconsistency 18](#_Toc155599469)

[Table S6: SUCRA values for all outcomes 21](#_Toc155599470)

[Table S7: CINeMA ratings for all comparisons of the seven outcomes 22](#_Toc155599471)

[Fig. S1: Risk of bias assessment for the included studies. 36](#_Toc155599472)

[Fig. S2: Network plot for pain freedom at 2 hours 37](#_Toc155599473)

[Fig. S3: Network plot for pain relief at 2 hours 37](#_Toc155599474)

[Fig. S4: Network plot for MBS freedom at 2 hours 38](#_Toc155599475)

[Fig. S5: Network plot for sustained pain freedom over 24 hours 38](#_Toc155599476)

[Fig. S6: Network plot for sustained pain relief over 24 hours 39](#_Toc155599477)

[Fig. S7: Network plot for freedom from photophobia at 2 hours 39](#_Toc155599478)

[Fig. S8: Network plot for freedom from phonophobia at 2 hours 40](#_Toc155599479)

[Fig. S9: Confidence in evidence for all drugs compared to placebo 40](#_Toc155599480)

[Reference list of included trials 41](#_Toc155599481)

# Table S1: Search strategy

| **PubMed database <Aug 31, 2023>** | | **Results** |
| --- | --- | --- |
| #1 | Migraine [MeSH Terms] OR “Migraine Disorders”[MeSH Terms] OR “Headache Disorders, Primary”[MeSH Terms] OR “migraine disorders”[Title/Abstract] OR “headache disorders, primary”[Title/Abstract] OR “migraine” [Title/Abstract] OR “migraineur” [Title/Abstract] OR “migraines” [Title/Abstract] OR “headache” [Title/Abstract] headaches [Title/Abstract] OR migrain* [Title/Abstract] | 14,892 |
| #2 | Rimegepant [MeSH Terms] OR Ubrogepant [MeSH Terms] OR Zavegepant [MeSH Terms] OR Lasmiditan [MeSH Terms] OR rimegepant [Title/Abstract] OR Nurtec [Title/Abstract] OR Nurtec ODT [Title/Abstract] OR BMS-927711 [Title/Abstract] OR ubrogepant [Title/Abstract] OR Ubrelvy [Title/Abstract] OR MK-1602 [Title/Abstract] OR zavegepant [Title/Abstract] OR Zavzpret [Title/Abstract] OR BHV-3500 [Title/Abstract] OR lasmiditan [Title/Abstract] OR Reyvow [Title/Abstract] OR Rayvow [Title/Abstract] OR LY573144 [Title/Abstract] OR COL-144 [Title/Abstract] | 168 |
| #3 | “Randomized Controlled Trial” [Publication Type] OR “Randomized Controlled Trials as Topic” [MeSH Terms] OR Random* [Title/Abstract] OR RCT* [Title/Abstract] OR Control* [Title/Abstract] | 2,202,289 |
| #4 | (Blind* [Title/Abstract]) OR (Singleblind* [Title/Abstract]) OR (Doubleblind* [Title/Abstract]) OR (Trebleblind* [Title/Abstract]) OR (Tripleblind* [Title/Abstract]) | 128,827 |
| #5 | (“phase 3 clinical trial” [Title/Abstract]) OR (“Phase-3 randomized controlled trials” [Title/Abstract]) OR (“phase 4 clinical trial” [Title/Abstract]) OR (“Phase-4 randomized controlled trials” [Title/Abstract]) OR (“Clinical Trial” [Title/Abstract]) OR (“Clinical Trials” [Title/Abstract]) OR (“Clinical Study” [Title/Abstract]) OR (“Clinical Studies” [Title/Abstract]) OR (“Intention to Treat Analysis” [Title/Abstract]) | 288,436 |
| #6 | #3 OR #4 OR #5 | 2,388,432 |
| #7 | #1 AND #2 AND #6 | 108 |
| **Cochrane Central Register of Controlled Trials** | |  |
| #1 | MeSH descriptor: [Migraine] explode all trees | 3,536 |
| #2 | MeSH descriptor: [Migraine Disorders] explode all trees | 3,536 |
| #3 | MeSH descriptor: [Headache Disorders, Primary] explode all trees | 4,000 |
| #4 | (“migraine disorders” or “headache disorders, primary” or “migraine” or “migraineur” or migraines or headache or headaches or migrain*): ti,ab,kw | 41,253 |
| #5 | #1 OR #2 OR #3 OR #4 | 41,254 |
| #6 | MeSH descriptor: [Rimegepant] explode all trees | - |
| #7 | MeSH descriptor: [Ubrogepant] explode all trees | - |
| #8 | MeSH descriptor: [Zavegepant] explode all trees | - |
| #9 | MeSH descriptor: [Lasmiditan] explode all trees | - |
| #10 | (rimegepant or Nurtec or “Nurtec ODT”or BMS-927711 or ubrogepant or Ubrelvy or MK-1602 or zavegepant or Zavzpret or BHV-3500 or lasmiditan or Reyvow or Rayvow or LY573144 or COL-144): ti,ab,kw | 438 |
| #11 | #6 OR #7 OR #8 OR #9 OR #10 | 438 |
| #12 | MeSH descriptor: [Randomized Controlled Trial] explode all trees | 25,733 |
| #13 | MeSH descriptor: [Randomized Controlled Trials as Topic] explode all trees | 47,401 |
| #14 | (Random* or RCT* or Control* or Blind* or Singleblind* or Doubleblind* Trebleblind* or Tripleblind*): ti,ab,kw | 1,561,469 |
| #15 | (“phase 3 clinical trial” or “Phase-3 randomized controlled trials” or “phase 4 clinical trial” or “Phase-4 randomized controlled trials” or “Clinical Trial” or “Clinical Trials” or “Clinical Study” or “Clinical Studies” or “Intention to Treat Analysis”): ti,ab,kw | 689,196 |
| #16 | #12 OR #13 OR #14 OR #15 | 1,595,900 |
| #17 | #5 AND #11 AND #16 | 384 |
| **EMBASE** | |  |
| #1 | 'Migraine (topic)'/exp OR 'Migraine Disorders (topic)'/exp OR 'Headache Disorders, Primary (topic)'/exp | 83,582 |
| #2 | 'migraine disorders' :ti,ab,kw OR 'headache disorders, primary' :ti,ab,kw OR 'migraine' :ti,ab,kw OR 'migraineur' :ti,ab,kw OR 'migraines' :ti,ab,kw OR 'headache' :ti,ab,kw OR 'headaches' :ti,ab,kw OR 'migrain*' :ti,ab,kw | 210,150 |
| #3 | #1 OR #2 | 226,511 |
| #4 | 'Rimegepant (topic)'/exp OR 'Ubrogepant (topic)'/exp OR 'Zavegepant (topic)'/exp OR 'Lasmiditan (topic)'/exp | 1,133 |
| #5 | 'rimegepant' :ti,ab,kw OR 'Nurtec' :ti,ab,kw OR 'Nurtec ODT' :ti,ab,kw OR BMS-927711 :ti,ab,kw OR 'ubrogepant' :ti,ab,kw OR 'Ubrelvy' :ti,ab,kw OR MK-1602 :ti,ab,kw OR 'zavegepant' :ti,ab,kw OR 'Zavzpret' :ti,ab,kw OR BHV-3500 :ti,ab,kw OR 'lasmiditan' :ti,ab,kw OR 'Reyvow' :ti,ab,kw OR 'Rayvow' :ti,ab,kw OR LY573144 :ti,ab,kw OR COL-144 :ti,ab,kw | 897 |
| #6 | #4 OR #5 | 1,178 |
| #7 | 'Clinical Trials, Phase II as Topic (topic)'/exp OR 'Clinical Trials, Phase III as Topic (topic)'/exp OR 'Clinical Trials, Phase IV as Topic (topic)'/exp OR 'Controlled Clinical Trials as Topic (topic)'/exp OR 'phase 3 clinical trial (topic)'/exp OR 'Phase-3 randomized controlled trials (topic)'/exp OR 'phase 4 clinical trial (topic)'/exp OR 'Phase-4 randomized controlled trials (topic)'/exp OR 'Randomized Controlled Trials as Topic (topic)'/exp OR 'Intention to Treat Analysis (topic)'/exp OR 'Randomized Controlled Trial (topic)'/exp OR 'Single-Blind Method (topic)'/exp OR 'Double-Blind Method (topic)'/exp | 1,187,831 |
| #8 | 'Clinical Trials, Phase II as Topic ':ti,ab,kw OR 'Clinical Trials, Phase III as Topic ':ti,ab,kw OR 'Clinical Trials, Phase IV as Topic ':ti,ab,kw OR 'Controlled Clinical Trials as Topic' :ti,ab,kw OR 'Randomized Controlled Trials as Topic' :ti,ab,kw OR ' Intention to Treat Analysis' :ti,ab,kw OR 'Pragmatic Clinical Trials as Topic' :ti,ab,kw OR 'Clinical Trials, Phase II' :ti,ab,kw OR 'Clinical Trials, Phase III' :ti,ab,kw OR 'Clinical Trials, Phase IV' :ti,ab,kw OR 'Controlled Clinical Trials' :ti,ab,kw OR 'Randomized Controlled Trials' :ti,ab,kw OR 'Pragmatic Clinical Trials as Topic' :ti,ab,kw OR 'Single-Blind Method' :ti,ab,kw OR 'Double-Blind Method' :ti,ab,kw 'randomized controlled trial' :ti,ab,kw OR random :ti,ab,kw OR 'random allocation' :ti,ab,kw OR 'clinical trials' :ti,ab,kw OR 'clinical trials as topic ' :ti,ab,kw OR 'clinical Study ' :ti,ab,kw | 1,139,229 |
| #9 | #7 OR #8 | 2,041,795 |
| #10 | #3 AND #6 AND #9 | 606 |
| **Web of Science** | |  |
| #1 | TS= (“Migraine” OR “Migraine Disorders” OR “Headache Disorders, Primary” OR “migraine disorders” OR “headache disorders, primary” OR “migraine” OR “migraineur” OR “migraines” OR Tobacco* OR “headache” OR “headaches” OR “migrain*”) | 497,551 |
| #2 | TI= (“Migraine” OR “Migraine Disorders” OR “Headache Disorders, Primary” OR “migraine disorders” OR “headache disorders, primary” OR “migraine” OR “migraineur” OR “migraines” OR Tobacco* OR “headache” OR “headaches” OR “migrain*”) | 152,212 |
| #3 | #1 OR #2 | 497,551 |
| #4 | TS= (Rimegepant OR Ubrogepant OR Zavegepant OR Lasmiditan OR rimegepant OR Nurtec OR “Nurtec ODT” OR BMS-927711 OR ubrogepant OR Ubrelvy OR MK-1602 OR zavegepant OR Zavzpret OR BHV-3500 OR Lasmiditan OR Reyvow OR Rayvow OR LY573144 OR COL-144) | 837 |
| #5 | TI= (Rimegepant OR Ubrogepant OR Zavegepant OR Lasmiditan OR rimegepant OR Nurtec OR “Nurtec ODT” OR BMS-927711 OR ubrogepant OR Ubrelvy OR MK-1602 OR zavegepant OR Zavzpret OR BHV-3500 OR Lasmiditan OR Reyvow OR Rayvow OR LY573144 OR COL-144) | 605 |
| #6 | #4 OR #5 | 837 |
| #7 | TS= (“randomized controlled trial” OR “Randomized Controlled Trials as Topic” OR “clinical trial” OR “single blind procedure” OR “controlled clinical trial” OR “double blind procedure” OR “phase 3 clinical trial” OR “Phase-3 randomized controlled trials” OR “phase 4 clinical trial” OR “Phase-4 randomized controlled trials”) | 790,216 |
| #8 | TI= (random* OR RCT OR control* OR blind* OR singleblind* OR doubleblind* OR trebleblind* OR tripleblind* OR “clinical trial” OR “clinical study” OR “intention to treat analysis” OR “phase 3 clinical trial” OR “Phase-3 randomized controlled trials” OR “phase 4 clinical trial” OR “Phase-4 randomized controlled trials”) | 2,411,335 |
| #9 | #7 OR #8 | 2,940,912 |
| #10 | #3 AND #6 AND #9 | 259 |

# Table S2: Detailed eligibility criteria for abstract and title screening

| Study design: | | |
| --- | --- | --- |
| - Randomised controlled trials | YES | NO |
| Participants/population: | | |
| - Adults (aged ≥18 years) with episodic or   chronic migraine with or without aura (where  specifed, International Headache Society  diagnostic criteria, Headache Classifcation  Committee of the International Headache  Society). | YES | NO |
| Intervention/comparators: | | |
| - Lasmiditan (50 mg, 100 mg, 200 mg)   Rimegepant (75 mg)  Ubrogepant (25 mg, 50 mg, 100 mg)  Zavegepant (5 mg, 10 mg, 20 mg) | YES | NO |
| Outcome: | | |
| - Pain freedom at 2 hours - Pain relief at 2 hours - Most bothersome symptom (MBS) freedom at 2 hours - Sustained pain freedom over 24 hours - Sustained pain relief over 24 hours - Freedom from photophobia at 2 hours - Freedom from phonophobia at 2 hours - Adverse events | YES | NO |
| Study inclusion: |  | |
| - All the answers are YES. | INCLUDE | |
| - Any answer is NO. | EXCLUDE | |

Instruction

1. We will include randomized controlled trial studies which provided the number of individuals treated and cured for each treatment arm (or number of treated individuals and cure rate).
2. Studies with two or more arms were included. Pairwise comparisons of drugs (lasmiditan, rimegepant, ubrogepant, zavegepant) were eligible. Studies comparing one or more of the aforementioned drugs with placebo were also eligible. In the case of articles with duplicate data, the one with more detailed data was included.
3. Conference proceedings, letters and editorials will be excluded.

# Table S3: List of excluded studies during full-text eligibility assessment

| **#** | **Study** | **Title** | **Reason for exclude** |
| --- | --- | --- | --- |
| 1 | Smith, 2021 | Randomized, Controlled Trial of Lasmiditan over Four Migraine Attacks: First Attack Finding | Only conference abstract |
| 2 | Tsai, 2021 | A Phase 1, Open-Label, Single-Dose Pharmacokinetic Study of Lasmiditan in Paediatric Patients with Migraine | Only conference abstract |
| 3 | Vincent, 2021 | Lasmiditan efficacy in mild versus moderate or severe migraine headaches | Only conference abstract |
| 4 | Nct, 2020 | Lasmiditan Compared to Placebo in the Acute Treatment of Migraine in Korean | Only conference abstract |
| 5 | Vargas, 2019 | Assessment to identify predictors of 2-hour pain freedom among patients enrolled in two phase 3 studies of lasmiditan for acute treatment of migraine | Only conference abstract |
| 6 | Schim, 2019 | Efficacy and safety of lasmiditan in patients on concomitant migraine preventive medications: findings from samurai and spartan phase 3 trials | Only conference abstract |
| 7 | Ashina, 2020 | Randomized, controlled trial of lasmiditan over four migraine attacks: consistency findings | Only conference abstract |
| 8 | He, 2021 | Lasmiditan over Four Migraine Attacks in Chinese Population: findings from CENTURION Study | Only conference abstract |
| 9 | Ctri, 2023 | A clinical study to assess the Efficacy and Safety of Lasmiditan Tablets compared to Placebo in Acute Treatment of Migraine with or Without Aura in Adult Patients | Only conference abstract |
| 10 | Rosen, 2020 | Long-term cardiovascular safety of lasmiditan for the acute treatment of migraine for up to one year: interim results of an open-label phase 3 study (Gladiator) | Only conference abstract |
| 11 | Rizzoli, 2019 | Safety findings from the phase 3 studies (samurai, spartan) of lasmiditan for acute treatment of migraine | Only conference abstract |
| 12 | Pearlman, 2019 | Effects of lasmiditan on driving performance: results of 2 randomized, blinded, crossover simulated driving studies with placebo and active controls | Only conference abstract |
| 13 | Clemow, 2020 | Effect of a change in lasmiditan dose on drug efficacy and safety: phase 3 study findings | Only conference abstract |
| 14 | Wilbraham, 2019 | A Randomized, Controlled, Crossover Study to Assess the Abuse Potential of Lasmiditan | Only conference abstract |
| 15 | Kudrow, 2019 | How Adverse Events Are Collected and Reported: Differences Between Randomized Phase 2 and Phase 3 Clinical Trials for Lasmiditan | Only conference abstract |
| 16 | Krege, 2019 | Safety findings from the phase 3 studies (SAMURAI, SPARTAN) of lasmiditan for acute treatment of migraine | Only conference abstract |
| 17 | Hochstetler, 2019 | Safety and efficacy of lasmiditan in patients with cardiovascular risk factors: results from two phase 3 trials for acute treatment of migraine | Only conference abstract |
| 18 | Doty, 2019 | Sustained efficacy of lasmiditan: results from phase 3 randomized clinical trials for acute treatment of migraine | Only conference abstract |
| 19 | Brandes, 2019 | Long-term safety and efficacy of lasmiditan for acute treatment of migraine over a one-year period: interim results of an open-label phase 3 study (gladiator) | Only conference abstract |
| 20 | Wietecha, 2018 | Phase 3 study (spartan) of lasmiditan compared to placebo for acute treatment of migraine | Only conference abstract |
| 21 | Wietecha, 2018 | Phase 3 studies (SAMURAI, SPARTAN) of lasmiditan compared to placebo for acute treatment of migraine | Only conference abstract |
| 22 | Kuca, 2017 | Lasmiditan (200 mg and 100 mg) compared to placebo for acute treatment of migraine | Only conference abstract |
| 23 | Euctr, 2017 | A clinical trial to study the safety of long term use of lasmiditan 100 mg and 200 mg in the treatment of migraine | Only conference abstract |
| 24 | Nct, 2015 | An Open-label, Long-term, Safety Study of Lasmiditan for the Acute Treatment of Migraine | Only conference abstract |
| 25 | Jensen, 2022 | Rimegepant for the Acute Treatment of Migraine: Subgroup Analyses From 3 Phase 3 Clinical Trials by Triptan Treatment Experience | Only conference abstract |
| 26 | L’ltalien, 2020 | Acute Treatment with Oral Rimegepant 75mg Reduces Migraine-Related Disability: Results from a One Year, Open-Label Safety Study (BHV3000-201) | Only conference abstract |
| 27 | Schim, 2021 | Rimegepant is Safe and Tolerable for the Acute Treatment of Migraine in Patients Using Preventive Migraine Medications: Results from a Long-Term Open-Label Safety Study | Only conference abstract |
| 28 | Lipton, 2022 | Effect of long-term preventive treatment with oral rimegepant 75 mg on migraine-related disability: A 52-week, open-label extension study | Only conference abstract |
| 29 | Lipton, 2021 | Onset of Migraine Preventive Effects With Rimegepant in a Phase 2/3, Randomized, Double-Blind, Placebo-Controlled Trial | Only conference abstract |
| 30 | Croop, 2020 | Rimegepant is Effective for the Acute Treatment of Migraine in Patients Who Have Discontinued or Currently Use Triptans: Results from 3 Phase 3 Clinical Trials | Only conference abstract |
| 31 | Jensen, 2021 | Rimegepant for the Acute Treatment of Migraine in Patients with a History of Triptan Treatment Failure: Pooled Results From 3 Phase 3 Clinical Trials | Only conference abstract |
| 32 | Croop, 2021 | A phase 2/3, randomized, double-blind, placebo-controlled study to evaluate the efficacy and safety of rimegepant for the preventive treatment of migraine | Only conference abstract |
| 33 | Popoff, 2022 | Acute treatment with rimegepant 75 mg offers improvements in pain severity over 48-hours when compared with placebo-Post hoc results from a randomized placebo-controlled trial (BHV3000-303) | Only conference abstract |
| 34 | Lipton, 2021 | Rapid decrease in migraine days with rimegepant: Results from a post hoc analysis of a phase 2/3, randomized, double-blind, placebo-controlled trial（下载了整个期刊） | Only conference abstract |
| 35 | Yu, 2022 | Efficacy, safety, and tolerability of rimegepant 75 mg orally disintegrating tablet for the acute treatment of migraine: Results from a phase 3, double-blind, randomized, placebo-controlled trial in adults from China and Korea | Only conference abstract |
| 36 | Li, 2022 | A phase 1, randomized, placebo-controlled, single-and multiple-dose, double-blind study to evaluate the pharmacokinetics and safety of rimegepant orally disintegrating tablets 75 mg in healthy Chinese adults | Only conference abstract |
| 37 | Johnston, 2022 | MSQ Utility Mapping of Rimegepant by Change in Monthly Migraine Days for Preventive Treatment of Migraine | Only conference abstract |
| 38 | Jensen, 2021 | Rimegepant for the Acute Treatment of Migraine: subgroup Analyses from 3 Phase 3 Clinical Trials by Number of Triptans Previously Tried and Failed | Only conference abstract |
| 39 | Turner, 2020 | Rimegepant 75 mg is more effective for migraine than nonsteroidal anti-inflammatory drugs: post hoc analysis of data from 2 phase 3 trials | Only conference abstract |
| 40 | Levin, 2020 | Rimegepant 75 mg is effective for the acute treatment of migraine regardless of attack frequency: results from 3 phase 3 trials | Only conference abstract |
| 41 | Buse, 2020 | Rimegepant 75 mg is safe and well tolerated for the acute treatment of migraine in adults with a history of depression or anxiety: results from a long-term open-label safety study (Study 201) | Only conference abstract |
| 42 | Nct, 2023 | A Study to Learn About the Safety and Effects of Rimegepant to Prevent Migraine in Chinese Subjects | Only conference abstract |
| 43 | Hutchinson, 2022 | Patterns of medication utilization and migraine frequency in adults using rimegepant for both preventive and acute treatment for migraine: results from a 52-week, open-label extension study | Only conference abstract |
| 44 | Smith, 2020 | Low rates of rescue medication usage in subjects treated with a single dose of rimegepant 75 mg for the acute treatment of migraine: results from 3 phase 3 clinical trials | Only conference abstract |
| 45 | Pavlovic, 2020 | Rimegepant is effective for the acute treatment of migraine in subjects taking concurrent preventive medication: results from 3 phase 3 trials | Only conference abstract |
| 46 | Turner, 2021 | Preference for rimegepant and improved clinical global impression of change among adults with a history of triptan treatment failure: results from a long-term open-label safety study | Only conference abstract |
| 47 | Pavlovic, 2019 | A single dose of rimegepant demonstrates sustained efficacy and low rescue medication use in the acute treatment of migraine: results from 3 phase 3 trials | Only conference abstract |
| 48 | Schim, 2020 | Rimegepant 75 mg demonstrates safety and tolerability similar to placebo with no effects of age, sex, or race in 3 phase 3 trials | Only conference abstract |
| 49 | Lipton, 2021 | Rimegepant for the acute treatment of migraine: subgroup analyses from 3 phase 3 clinical trials by number of triptans previously tried and failed | Only conference abstract |
| 50 | Pavlovic, 2023 | Long-Term Preventive and Acute Treatment of Migraine With Rimegepant Improves Health Related Quality of Life | Only conference abstract |
| 51 | Croop, 2022 | Phase 2/3, randomized, double-blind, placebo-controlled study evaluating rimegepant for the preventive treatment of migraine | Only conference abstract |
| 52 | Mullin, 2023 | Medication Preference, Satisfaction, and Clinical Improvement Among Adults Receiving Long Term Treatment With Rimegepant for Migraine | Only conference abstract |
| 53 | Jensen, 2022 | Rimegepant for the acute treatment of migraine with and without a history of triptan failure | Only conference abstract |
| 54 | Lipton, 2023 | Safety and Tolerability of Rimegepant Every Other Day for Preventive Treatment of Migraine Plus As-Needed for Acute Treatment of Migraine: results from A 52-Week, Open-Label Extension Phase | Only conference abstract |
| 55 | Chaeleston, 2023 | Efficacy and Safety of Rimegepant forthe Acute Treatment of Migraine in Black and African American Adults: results From 3 Randomized, Placebo-Controlled Clinical Trials | Only conference abstract |
| 56 | Croop, 2023 | Effects of Rimegepant 75 mg on Monthly Migraine Days: a 52-Week, Open-Label Extension Study | Only conference abstract |
| 57 | McAllister, 2020 | Rimegepant 75 mg demonstrates superiority to placebo on nausea freedom: results from a post hoc pooled analysis of 3 phase 3 trials in the acute treatment of migraine | Only conference abstract |
| 58 | Pavlovic, 2020 | Rimegepant 75 mg provides early and sustained relief of migraine with a single oral dose: Results from 3 phase 3 clinical trials | Only conference abstract |
| 59 | Ailani, 2023 | Effects of Rimegepant 75 mg on Monthly Migraine Days: a 52-Week, Open-Label Extension Study | Only conference abstract |
| 60 | Mullin, 2021 | Rimegepant 75 mg for the acute treatment of migraine in adults with frequent migraine: Long-term safety and clinical improvement versus baseline | Only conference abstract |
| 61 | Croop, 2020 | Long-term safety of rimegepant 75 mg for the acute treatment of migraine (Study 201) | Only conference abstract |
| 62 | Turner, 2021 | Acute treatment of migraine with rimegepant improves health related quality of life in adults with a history of triptan treatment failure: Results from a long-term, open-label safety study | Only conference abstract |
| 63 | Hutchinson, 2020 | Oral rimegepant 75 mg is safe and well tolerated in adults with migraine and cardiovascular risk factors: Results of a multicenter, long-term, open-label safety study | Only conference abstract |
| 64 | Turner, 2020 | Patient preference and improved clinical global impression of change with rimegepant 75 mg for the acute treatment of migraine: Results from a long-term open-label safety study (Study 201) | Only conference abstract |
| 65 | Baskin, 2020 | Rimegepant 75 mg is safe and well tolerated for the acute treatment of migraine in adults using selective serotonin reuptake inhibitors (SSRIS) and other antidepressants: Results from a long-term, open-label safety study (Study 201) | Only conference abstract |
| 66 | MacAllister, 2019 | Rimegepant 75mg demonstrates superiority to placebo on nausea freedom: results from a post hoc pooled analysis of 3 phase 3 trials in the acute treatment of migraine | Only conference abstract |
| 67 | Lipton, 2019 | A single dose of rimegepant 75 mg provides pain relief and return to normal function: results from 3 phase 3 trials in adults with migraine | Only conference abstract |
| 68 | Lipton, 2019 | Efficacy, Safety, and Tolerability of Rimegepant 75 mg Orally Dissolving Tablet for the Acute Treatment of Migraine: Results from a Phase 3, Double-Blind, Randomized, Placebo-Controlled Trial, Study 303 | Only conference abstract |
| 69 | Lipton, 2019 | Long-term, open-label safety study of rimegepant 75 mg for the treatment of migraine (study 201): interim analysis of safety and exploratory efficacy | Only conference abstract |
| 70 | Lipton, 2019 | Rimegepant 75mg provides pain relief and return to normal function with a single dose: results from 3 phase 3 trials in adults with migraine | Only conference abstract |
| 71 | Hutchinson, 2019 | Safety of rimegepant 75 mg in adults with migraine: no effects of age, sex, or race in 3 phase 3 trials | Only conference abstract |
| 72 | Hutchinson, 2019 | The safety and tolerability of rimegepant 75 mg are similar to placebo: results from 3 phase 3 trials in adults with migraine | Only conference abstract |
| 73 | Hutchinson, 2019 | Rimegepant 75mg demonstrates safety and tolerability similar to placebo: results from 3 phase 3 trials in adults with migraine | Only conference abstract |
| 74 | Dodick, 2019 | Rimegepant is effective for the acute treatment of migraine in subjects taking concurrent preventive medication: results from 3 phase 3 trials | Only conference abstract |
| 75 | Croop, 2019 | Rimegepant 75 mg demonstrates superiority to placebo on nausea freedom: results from a post hoc pooled analysis of 3 phase 3 trials in the acute treatment of migraine | Only conference abstract |
| 76 | Buse, 2019 | Rimegepant 75mg is effective for the acute treatment of migraine regardless of attack frequency: results from 3 phase 3 trials | Only conference abstract |
| 77 | Blumenfeld, 2019 | Rimegepant 75 mg is more effective than nonsteroidal anti-inflammatory drugs for the acute treatment of migraine: post hoc analysis of data from 2 phase 3 trials | Only conference abstract |
| 78 | Lipton, 2018 | Rimegepant 75 MG, an oral calcitonin gene-related peptide antagonist, for the acute treatment of migraine: two phase 3, double-blind, randomized, placebo-controlled trials | Only conference abstract |
| 79 | Lipton, 2018 | Efficacy, Safety, and Tolerability of Rimegepant 75 Mg, an Oral CGRP Receptor Antagonist, for the Acute Treatment of Migraine: Results from a Double-blind, Randomized, Placebo-controlled Trial, Study 301 | Only conference abstract |
| 80 | Krege, 2022 | Lasmiditan for Patients with Migraine and Contraindications to Triptans: A Post Hoc Analysis | Unrelated study design |
| 81 | Takeshima, 2022 | Efficacy of Lasmiditan Across Patient and Migraine Characteristics in Japanese Patients with Migraine: A Secondary Analysis of the MONONOFU Trial | Unrelated study design |
| 82 | Matsumori, 2022 | Rapid Onset and Sustained Efficacy of Lasmiditan Among Japanese Patients with Migraine: Prespecified Analyses of a Randomized Controlled Trial | Unrelated study design |
| 83 | Ashina, 2023 | Long-term treatment with lasmiditan in patients with migraine: Results from the open-label extension of the CENTURION randomized trial | Unrelated outcome |
| 84 | Shapiro, 2019 | Lasmiditan for acute treatment of migraine in patients with cardiovascular risk factors: post-hoc analysis of pooled results from 2 randomized, double-blind, placebo-controlled, phase 3 trials | Unrelated study design |
| 85 | Kitamura, 2023 | Lasmiditan in Japanese Patients with Common Migraine Comorbidities or Concomitant Medications: A Post Hoc Safety and Efficacy Analysis from the MONONOFU Study | Unrelated study design |
| 86 | Hashimoto, 2023 | Lasmiditan for single migraine attack in Japanese patients with cardiovascular risk factors: subgroup analysis of a phase 2 randomized placebo-controlled trial | Unrelated study design |
| 87 | Clemow, 2020 | Lasmiditan in patients with common migraine comorbidities: aposthoc efficacy and safety analysis of two phase 3 randomized clinical trials | Unrelated study design |
| 88 | Peres, 2021 | Lasmiditan efficacy in migraine attacks with mild vs. moderate or severe pain | Unrelated study design |
| 89 | Hirata, 2023 | Safety profile of lasmiditan in patients with migraine in an Asian population | Unrelated study design |
| 90 | Loo, 2019 | Effect of a rescue or recurrence dose of lasmiditan on efficacy and safety in the acute treatment of migraine: findings from the phase 3 trials (SAMURAI and SPARTAN) | Unrelated study design |
| 91 | Loo, 2019 | Efficacy and safety of lasmiditan in patients using concomitant migraine preventive medications: findings from SAMURAI and SPARTAN, two randomized phase 3 trials | Unrelated study design |
| 92 | Krege, 2019 | Safety findings from Phase 3 lasmiditan studies for acute treatment of migraine: Results from SAMURAI and SPARTAN | Unrelated study design |
| 93 | Doty, 2091 | Sustained responses to lasmiditan: Results from post-hoc analyses of two Phase 3 randomized clinical trials for acute treatment of migraine | Unrelated study design |
| 94 | Ashina, 2019 | Onset of Efficacy Following Oral Treatment With Lasmiditan for the Acute Treatment of Migraine: integrated Results From 2 Randomized Double‐Blind Placebo‐Controlled Phase 3 Clinical Studies | Unrelated study design |
| 95 | Farkkila, 2012 | Acute treatment of migraine with the selective 5-HT1F receptor agonist lasmiditan - A randomised proof-of-concept trial | Unrelated outcome |
| 96 | Berman, 2020 | Safety of Rimegepant, an Oral CGRP Receptor Antagonist, Plus CGRP Monoclonal Antibodies for Migraine | Unrelated intervention |
| 97 | Ashina, 2021 | Randomized, controlled trial of lasmiditan over four migraine attacks: Findings from the CENTURION study | Unrelated outcome |
| 98 | Croop, 2021 | Oral rimegepant for preventive treatment of migraine: a phase 2/3, randomised, double-blind, placebo-controlled trial | Unrelated outcome |
| 99 | Gao, 2020 | Efficacy and Safety of Rimegepant for the Acute Treatment of Migraine: Evidence From Randomized Controlled Trials | Unrelated study design |
| 100 | Bertz, 2023 | A placebo-controlled, randomized, single and multiple dose study to evaluate the safety, tolerability, and pharmacokinetics of rimegepant in healthy participants | Unrelated outcome |
| 101 | Johnson, 2022 | Health state utility mapping of rimegepant for the preventive treatment of migraine: BHV3000-305 double blind treatment phase and open label extension study | Unrelated study design |
| 102 | Li, 2021 | Pharmacokinetics and Safety of Single and Multiple Daily Dosing of 75-mg Rimegepant Orally Disintegrating Tablets in Healthy Chinese Adults: A Randomized Placebo-Controlled Trial | Unrelated outcome |
| 103 | Bhardwaj, 2023 | Rimegepant 75 mg in Subjects With Hepatic Impairment: Results of a Phase 1, Open-Label, Single-Dose, Parallel-Group Study | Unrelated outcome |
| 104 | Lipton, 2023 | Efficacy of rimegepant for the acute treatment of migraine based on triptan treatment experience: Pooled results from three phase 3 randomized clinical trials | Unrelated study design |
| 105 | Johnston, 2021 | Mapping Migraine-Specific Quality of Life to Health State Utilities in Patients Receiving Rimegepant | Unrelated outcome |
| 106 | L’ltalien, 2021 | Rimegepant 75 mg for acute treatment of migraine is associated with significant reduction in monthly migraine days: Results from a long-term, open-label study | Unrelated outcome |
| 107 | Popoff, 2021 | Matching-adjusted indirect comparisons of oral rimegepant versus placebo, erenumab, and galcanezumab examining monthly migraine days and health-related quality of life in the treatment of migraine | Unrelated intervention |
| 108 | Johnston, 2022 | Monthly migraine days, tablet utilization, and quality of life associated with Rimegepant – post hoc results from an open label safety study (BHV3000–201) | Unrelated study design |
| 109 | McCarthy, 2019 | Oral rimegepant increased freedom from pain and from most bothersome symptom at 2 h in acute migraine | Unrelated study design |
| 110 | Hutchinson, 2021 | Ubrogepant for the Acute Treatment of Migraine: Pooled Efficacy, Safety, and Tolerability From the ACHIEVE I and ACHIEVE II Phase 3 Randomized Trials | Unrelated study design |
| 111 | Dodick, 2020 | Ubrogepant, an Acute Treatment for Migraine, Improved Patient-Reported Functional Disability and Satisfaction in 2 Single-Attack Phase 3 Randomized Trials, ACHIEVE I and II | Unrelated study design |
| 112 | Hutchinson, 2020 | Ubrogepant for the acute treatment of migraine: pooled efficacy from achieve ii and achieve ii phase 3 trials | Unrelated study design |
| 113 | Ailani, 2020 | Long‐Term Safety Evaluation of Ubrogepant for the Acute Treatment of Migraine: phase 3, Randomized, 52‐Week Extension Trial | Unrelated study design |
| 114 | Blumenfeld, 2022 | Ubrogepant Is Safe and Efficacious in Participants Taking Concomitant Preventive Medication for Migraine: A Pooled Analysis of Phase 3 Trials | Unrelated study design |
| 115 | Chiang, 2021 | Real world efficacy, tolerability and safety of ubrogepant | Unrelated study design |
| 116 | Trugman, 2019 | Safety an Tolerability of Ubrogepant Following Intermittent, High Frequency Dosing | Unrelated study design |
| 117 | Reuter, 2021 | Lasmiditan is Effective in the Acute Treatment of Migraine in Patients with Insufficient Response to Triptans: Findings from the Modified-parallel, Placebo-controlled, Double-blind, Phase 3 Consistency CENTURION Study | Only conference abstract |
| 118 | Lipton, 2021 | Intranasal Zavegepant is Effective and Well Tolerated for the Acute Treatment of Migraine: A Phase 2/3 Dose-Ranging Clinical Trial | Unrelated outcome |
| 119 | Viswanathan, 2019 | Improved Functionality, Pain Relief, and Satisfaction in Patients Treated With Ubrogepant vs Placebo: Results from 2 Single-Attack Phase 3 Studies, ACHIEVE I and II | Unrelated outcome |
| 120 | Pavlovic, 2021 | Ubrogepant was safe andwell tolerated in the acute treatment of perimenstrual migraine | Unrelated outcome |
| 121 | Dodick, 2023 | Ubrogepant for the Acute Treatment of Migraine When Administered During the Prodrome (Premonitory Phase): results From a Phase 3, Randomized, Double-blind, Placebo-Controlled, Crossover Study | Unrelated outcome |
| 122 | Lipton, 2023 | Improvement in Patient-Reported Outcomes When Ubrogepant Is Administered During the Migraine Prodrome (Premonitory Phase): results From the PRODROME Trial | Unrelated study design |
| 123 | Goadsby, 2023 | Efficacy of Ubrogepant for the Treatment of Migraine Symptoms During the Prodrome (Premonitory Phase): results From the PRODROME Trial | Unrelated study design |
| 124 | Grozeva, 2022 | Comparative Bioavailability of Single-Dose Zavegepant Nasal Spray During and Between Migraine Attacks: A Phase 1, Randomized, Open-Label, Fixed-Sequence, 2-Period Study | Unrelated study design |
| 125 | Lipton, 2019 | Efficacy, safety, and tolerability of ubrogepant for the acute treatment of migraine: a single-attack phase 3 study, ACHIEVE II | Duplicate data source |
| 126 | Hutchinson, 2019 | Ubrogepant for the acute treatment of migraine: pooled safety and tolerability from achieve i and achieve ii phase 3 studies | Unrelated outcome |
| 127 | Dodick, 2019 | Improved Functionality, Pain Relief, and Satisfaction in Patients Treated with Ubrogepant vs Placebo: results from 2 Single-Attack Phase 3 Studies, ACHIEVE i and II | Unrelated outcome |
| 128 | Dodick, 2019 | Ubrogepant achieves onset of pain relief at 1 hour for the acute treatment of migraine | Unrelated outcome |
| 129 | Trugman, 2018 | Efficacy, safety, and tolerability of ubrogepant for the acute treatment of migraine: results from a single-attack phase II study, ACHIEVE i | Unrelated outcome |
| 130 | Dodick, 2018 | Evaluating the impact of ubrogepant, an acute treatment for migraine, on patient-reported functionality and satisfaction: results from a single attack phase iii study, ACHIEVE i | Duplicate data source |
| 131 | Nct, 2016 | An Extension Study to Evaluate the Long-Term Safety and Tolerability of Ubrogepant in the Treatment of Migraine | Unrelated outcome |
| 132 | Goadsby, 2019 | Safety and tolerability of ubrogepant following intermittent, high-frequency dosing: randomized, placebo-controlled trial in healthy adults | Unrelated outcome |
| 133 | Lipton, 2016 | A phase 2b randomized, double-blind, placebo-controlled trial of ubrogepant for acute treatment of a migraine attack | Unrelated outcome |
| 134 | Cipolla, 2021 | Zavegepant Calcitonin gene-related peptide (CGRP) receptor antagonist Treatment of migraine | Unrelated outcome |
| 135 | Croop, 2021 | Intranasal Zavegepant is Effective and Well Tolerated for the Acute Treatment of Migraine: A Phase 2/3 Dose-Ranging Clinical trial | Unrelated outcome |
| 136 | Tassorelli, 2021 | Safety Findings from CENTURION, a Phase 3 Consistency Study of Lasmiditan for the Acute Treatment of Migraine | Only conference abstract |
| 137 | Mullin, 2023 | Efficacy and Safety of Zavegepant Nasal Spray for the Acute Treatment of Migraine: results of a Phase 3 Double-Blind, Randomized, Placebo Controlled Trial | Duplicate data source |
| 138 | Berta, 2022 | Comparative Bioavailability of Single-Dose Zavegepant Nasal Spray During and Between Migraine Attacks: a Phase 1, Randomized, Open-Label, Fixed-Sequence, 2-Period Study | Unrelated outcome |
| 139 | Berta, 2022 | Safety, Tolerability, and Pharmacokinetics of Single and Multiple Ascending Doses of Intranasal Zavegepant in Healthy Adults | Unrelated outcome |
| 140 | Berta, 2023 | Concentration QT Interval Modeling of Intranasally Administered Zavegepant in Healthy Subjects | Unrelated outcome |
| 141 | Berta, 2022 | Effects of zavegepant and concomitant sumatriptan on blood pressure and pharmacokinetics in healthy adult participants | Unrelated outcome |

# Table S4: Basic characteristics of included studies

| **Study** | **Country** | **Phase** | **Patient source** | **Intervention** | **Sample** | **Gender M/F** | **Age**  **Mean ± SD** | **Follow-up** | **Outcome** | **Diagnostic criteria** |
| --- | --- | --- | --- | --- | --- | --- | --- | --- | --- | --- |
| Sakai 2020^1^ | Japan | phase 2 | patients from 34 sites in Japan | Lasmiditan 50mg  Lasmiditan 100mg  Lasmiditan 200mg  Placebo | 87  208  182  214 | 42/45  32/176  37/145  36/178 | 44.9 ± 10.2  45.7 ± 9.7  44.7 ± 10.4  45.2 ± 9.0 | 4 weeks | ①②③④ | participants with at least a 1-year history of migraine with or without aura (IHS criteria 1.1 or 1.2.1) |
| Yu  2022^2^ | China | phase 3 | multicenter | Lasmiditan 100 mg  Lasmiditan 200 mg  Placebo | 95 92 94 | 25/70  24/68  29/65 | 37.5 ± 9.7  37.5 ± 9.5  38.3 ± 10.1 | / | ①② | participants with at least a 1-year history of migraine with or without aura (IHS criteria 1.1 or 1.2.1) |
| Brandes 2020^3^ | USA | phase 3 | patients from 199 study sites in the US, UK, and Germany | Lasmiditan 100 mg  Lasmiditan 200 mg | 991  1039 | 298/1732 | 43.3  range(18-79 ) | / | ① | participants with at least a 1-year history of migraine with or without aura (IHS criteria 1.1 or 1.2.1) |
| Goadsby 2019^4^ | USA | phase 3 | patients from 125 headache centres in the USA, UK and Germany | Lasmiditan 50 mg  Lasmiditan 100 mg  Lasmiditan 200 mg  Placebo | 654  635  649  645 | 100/554  96/539  113/536  100/545 | 42.8 ±13.2  43.4 ± 12.6  41.8 ± 12.4  42.6 ± 12.9 | 1 week | ①②③④⑥⑦ | participants with at least a 1-year history of migraine with or without aura (IHS criteria 1.1 or 1.2.1) |
| Brandes 2019^5^ | USA | phase 3 | patients from 199 study sites in the US, UK, and Germany | Lasmiditan 100 mg  Lasmiditan 200 mg | 963  1015 | 141/822  149/866 | 42.7 ± 12.3  43.8 ±12.5 | / | ① | participants with at least a 1-year history of migraine with or without aura (ICHD-II) |
| Kuca 2018^6^ | USA | phase 3 | patients from study site personnel at 99 centers in the US | Lasmiditan 100mg  Lasmiditan 200mg  Placebo | 630  609  617 | 118/512  94/515  92/525 | 42.2 ± 11.7  41.4 ± 12.0  42.4 ± 12.3 | 48 hours | ①③④⑥⑦ | participants with at least a 1-year history of migraine with or without aura (IHS criteria 1.1 or 1.2.1) |
| Farkkila 2012^7^ | Finland | phase 2 | patients from 43 headache centres in five European countries | Lasmiditan 50mg  Lasmiditan 100mg  Lasmiditan 200mg  Placebo | 82  83  71  86 | 13/69  14/69  6/65  11/75 | 40.4 ± 12.5  42.0 ± 10.6  39.5 ± 10.3  40.5 ± 10.3 | 2 weeks | ① | participants with at least a 1-year history of migraine with or without aura (IHS criteria 1.1 or 1.2.1) |
| Ashina 2021^8^ | USA | phase 3 | multicenter, conducted in Europe, North America, and Asia | Lasmiditan 100mg  Lasmiditan 200mg  Placebo | 485  486  500 | 402/83  400/86  417/83 | 42 ± 12  42 ± 12  41 ± 12 | / | ①②③④ | participants with at least a 1-year history of migraine with or without aura (IHS criteria 1.1 or 1.2.1) |
| Yu 2023^9^ | China | phase 3 | patients rom 86 study centres (outpatient clinics at hospitals and academic medical centres, 73 in China and 13 in South Korea) | Rimegepant 75 mg  Placebo | 666  674 | 141/525  111/563 | 37  range(30–45)  36  range(30–44) | 1 week | ①②③④ | participants with at least a 1-year history of migraine with or without aura (ICHD-3-beta) |
| Lipton 2019(1)^10^ | UK | phase 3 | multicenter | Rimegepant 75 mg  Placebo | 537  535 | 58/479  63/472 | 40.2 ± 11.9  40.9 ± 12.1 | 4 weeks | ①②③④⑤⑥⑦ | participants with at least a 1-year history of migraine with or without aura (ICHD-3-beta) |
| Croop 2019^11^ | USA | phase 3 | patients from 69 study centres in the USA | Rimegepant 75 mg  Placebo | 669  682 | 101/568  103/579 | 40.3 ± 12.1  40.0 ± 11.9 | 45 days | ①②③④⑤⑥⑦ | participants with at least a 1-year history of migraine with or without aura (ICHD-3-beta) |
| Marcus 2014^12^ | USA | phase 2 | patients from multicenter outpatient clinics | Rimegepant 75 mg  Placebo | 170  282 | 89/81  86/196 | 38.5 ± 11.87  37.9 ± 11.36 | 1 week | ①④⑤⑥⑦ | migraine headache at least a 1-year history of migraine with or without aura |
| Lipton 2022^13^ | USA | phase 3 | multicenter | Ubrogepant 50 mg  Ubrogepant 100 mg | 401  407 | 31/370  43/364 | 42.4±12.2  41.5±11.2 | 48 hours | ①⑥⑦ | migraine with or without aura |
| Lipton 2019(2)^14^ | USA | phase 3 | patients from 99 study centers (primary care and research clinics) in the US | Ubrogepant 50 mg  Ubrogepant 25 mg  Placebo | 488  478  499 | 44/444  47/431  57/442 | 41.2 ± 12.5  41.6 ± 12.4  41.7 ± 12.1 | 4 weeks | ①②③④⑤⑥⑦ | participants with at least a 1-year history of migraine with or without aura (ICHD-3-beta) |
| Dodick 2019^15^ | USA | phase 3 | patients from 89 centers in the US | Ubrogepant 50 mg  Ubrogepant 100 mg  Placebo | 466  485  485 | 48/418  67/418  55/430 | 40.1 ±11.7  40.6 ± 12.0  40.9 ± 11.7 | 4 weeks | ①②③④⑤⑥⑦ | participants with at least a 1-year history of migraine with or without aura (ICHD-3-beta) |
| Voss 2016^16^ | USA | phase IIb | multicenter | Ubrogepant 25 mg  Ubrogepant 50 mg  Ubrogepant 100 mg  Placebo | 104  106  102  113 | 13/91  14/92  12/90  14/99 | 41.4 ± 11.5  40.7 ± 12.3  41.9 ± 11.0  40.5 ± 11.7 | 48 hours | ①④⑤⑥⑦ | participants with at least a 1-year history of migraine with or without aura (ICHD-II) |
| Croop 2022^17^ | USA | phase 2/3 | patients from academic medical centers, private practices, and independent research facilities | Zavegepant 5mg  Zavegepant 10 mg  Zavegepant 20 mg  Placebo | 387  391  402  401 | 51/336  58/333  58/344  63/338 | 41.9 ± 12.6  41.4 ± 12.9  40.0 ± 13.0  39.9 ±12.0 | 1 week | ②④⑤⑥⑦ | participants with at least a 1-year history of migraine with or without aura (ICHD-3-beta) |
| Lipton 2023^18^ | USA | phase 3 | patients from 90 study centres (academic medical centres, headache clinics, and independent research facilities) in the USA | Zavegepant 10 mg  Placebo | 623  646 | 117/506  100/546 | 40.9 ± 13.2  40.8 ± 13.5 | 1 week | ①②③④⑤⑥⑦ | participants with at least a 1-year history of migraine with or without aura (ICHD-3-beta) |

M:male; F: female; IHS: International Headache Society; ICHD-II: International Classification of Headache Disorders, second edition criteria; ICHD-3-beta: International Classification of Headache Disorders Criteria, 3rd edition (beta version); ① pain freedom at 2 hours; ② pain relief at 2 hours; ③ most bothersome symptom (MBS) freedom at 2 hours; ④ sustained pain freedom over 24 hours; ⑤ sustained pain relief over 24 hours; ⑥ freedom from photophobia at 2 hours; ⑦ freedom from phonophobia at 2 hours.

# Table S5: Node splitting test for inconsistency

| **Outcome** | **Side** | **Direct** | |  | **Indirect** | |  | **Difference** | | ***P* > \|z\|** |
| --- | --- | --- | --- | --- | --- | --- | --- | --- | --- | --- |
|  |  | **Coef.** | **Std. Err.** |  | **Coef.** | **Std. Err.** |  | **Coef.** | **Std. Err.** |  |
| Pain freedom at 2 hours | B vs. C | 0.181 | 0.937 |  | 0.188 | 44.774 |  | -0.007 | 44.775 | 1.000 |
|  | B vs. A | -0.151 | 0.184 |  | -0.237 | 0.341 |  | 0.086 | 0.378 | 0.820 |
|  | B vs. K | -0.425 | 0.125 |  | -0.410 | 0.408 |  | -0.016 | 0.421 | 0.971 |
|  | C vs. A | -0.386 | 0.178 |  | -0.210 | 0.335 |  | -0.176 | 0.368 | 0.633 |
|  | C vs. K | -0.604 | 0.124 |  | -0.620 | 0.408 |  | 0.016 | 0.421 | 0.971 |
|  | A vs. K | -0.277 | 0.186 |  | -0.145 | 0.400 |  | -0.132 | 0.442 | 0.764 |
|  | K vs. D | 0.595 | 0.154 |  | 0.755 | 84.711 |  | -0.160 | 84.711 | 0.998 |
|  | K vs. G | 0.715 | 0.234 |  | 0.528 | 0.364 |  | 0.187 | 0.435 | 0.667 |
|  | K vs. E | 0.514 | 0.233 |  | 0.303 | 0.621 |  | 0.211 | 0.678 | 0.756 |
|  | K vs. F | 0.515 | 0.181 |  | 0.566 | 0.626 |  | -0.051 | 0.644 | 0.936 |
|  | K vs. I | 0.462 | 0.259 |  | 0.795 | 130.004 |  | -0.332 | 130.004 | 0.998 |
|  | G vs. E | -0.185 | 0.346 |  | -0.169 | 0.291 |  | -0.017 | 0.452 | 0.971 |
|  | G vs. F | -0.134 | 0.163 |  | -0.259 | 0.603 |  | 0.125 | 0.627 | 0.842 |
|  | E vs. F | 0.025 | 0.215 |  | 0.082 | 0.533 |  | -0.057 | 0.574 | 0.921 |
| Pain relief at 2 hours | B vs. C | -0.007 | 0.026 |  | 0.445 | 19.834 |  | -0.452 | 19.834 | 0.982 |
|  | B vs. A | -0.109 | 0.038 |  | -0.138 | 0.097 |  | 0.029 | 0.100 | 0.770 |
|  | B vs. K | -0.362 | 0.034 |  | -0.136 | 9.979 |  | -0.226 | 9.979 | 0.982 |
|  | C vs. A | -0.098 | 0.038 |  | -0.159 | 0.097 |  | 0.060 | 0.100 | 0.546 |
|  | C vs. K | -0.355 | 0.034 |  | 0.097 | 20.025 |  | -0.452 | 20.025 | 0.982 |
|  | A vs. K | -0.229 | 0.046 |  | -0.382 | 0.012 |  | 0.153 | 0.131 | 0.241 |
|  | K vs. D | 0.032 | 0.030 |  | 0.739 | 28.524 |  | -0.423 | 28.525 | 0.988 |
|  | K vs. G | 0.223 | 0.061 |  | 0.033 | 0.156 |  | -0.103 | 0.173 | 0.552 |
|  | K vs. E | 0.226 | 0.062 |  | 0.123 | 0.156 |  | 0.103 | 0.173 | 0.552 |
|  | K vs. F | 0.227 | 0.043 |  | 0.646 | 38.767 |  | -0.409 | 38.767 | 0.992 |
|  | K vs. I | 0.149 | 0.040 |  | 0.698 | 39.168 |  | -0.549 | 39.169 | 0.989 |
|  | K vs. J | 0.132 | 0.061 |  | 0.222 | 0.145 |  | -0.090 | 0.161 | 0.579 |
|  | K vs. H | 0.077 | 0.064 |  | 0.166 | 0.147 |  | -0.090 | 0.161 | 0.579 |
|  | G vs. F | -0.012 | 0.054 |  | 0.091 | 0.163 |  | -0.103 | 0.173 | 0.552 |
|  | E vs. F | 0.037 | 0.053 |  | -0.066 | 0.166 |  | 0.103 | 0.173 | 0.522 |
|  | I vs. J | 0.010 | 0.057 |  | -0.080 | 0.150 |  | 0.090 | 0.161 | 0.579 |
|  | I vs. H | -0.046 | 0.060 |  | -0.136 | 0.151 |  | 0.090 | 0.161 | 0.579 |
|  | J vs. H | - | - |  | - | - |  | - | - | - |
| MBS freedom at 2 hours | B vs. C | 0.029 | 0.068 |  | -0.024 | 35.14 |  | 0.053 | 35.14 | 0.999 |
|  | B vs. A | -0.095 | 0.112 |  | 0.033 | 0.225 |  | -0.128 | 0.245 | 0.601 |
|  | B vs. K | -0.155 | 0.072 |  | -0.181 | 17.563 |  | 0.027 | 17.563 | 0.999 |
|  | C vs. A | -0.126 | 0.112 |  | 0.162 | 0.224 |  | -0.143 | 0.0245 | 0.560 |
|  | C vs. K | -0.183 | 0.071 |  | -0.236 | 35.225 |  | 0.053 | 35.225 | 0.999 |
|  | A vs. K | -0.142 | 0.103 |  | 0.183 | 0.212 |  | -0.325 | 0.234 | 0.166 |
|  | K vs. D | 0.336 | 0.079 |  | 0.283 | 49.291 |  | 0.053 | 49.291 | 0.999 |
|  | K vs. G | 0.307 | 0.152 |  | 0.347 | 0397 |  | -0.041 | 0.430 | 0.925 |
|  | K vs. E | 0.218 | 0.155 |  | 0.178 | 0.398 |  | 0.041 | 0.430 | 0.925 |
|  | K vs. F | 0.339 | 0.104 |  | 0.229 | 61.741 |  | 0.111 | 61.741 | 0.999 |
|  | K vs. I | 0.242 | 0.135 |  | 0.299 | 85.224 |  | -0.057 | 85.224 | 0.999 |
|  | G vs. F | 0.022 | 0.146 |  | 0.063 | 0.405 |  | -0.041 | 0.430 | 0.925 |
|  | E vs. F | 0.131 | 0.147 |  | 0.091 | 0.407 |  | 0.041 | 0.430 | 0.925 |
| Sustained pain freedom over 24 hours | B vs. C | 0.159 | 0.347 |  | 0.434 | 40.257 |  | -0.275 | 40.259 | 0.995 |
|  | B vs. A | -0.161 | 0.523 |  | -0.279 | 1.148 |  | 0.117 | 1.253 | 0.925 |
|  | B vs. K | -0.5004 | 0.353 |  | -0.363 | 20.128 |  | -0.137 | 20.131 | 0.995 |
|  | C vs. A | -0.351 | 0.522 |  | -0.284 | 1.147 |  | -0.068 | 1.252 | 0.957 |
|  | C vs. K | -0.66 | 0.352 |  | -0.385 | 40.253 |  | -0.275 | 40.254 | 0.995 |
|  | A vs. K | -0.312 | 0.528 |  | -0.363 | 1.158 |  | 0.051 | 1.270 | 0.968 |
|  | K vs. D | 0.126 | 0.355 |  | 0.448 | 47.988 |  | -0.322 | 47.990 | 0.995 |
|  | K vs. G | 0.869 | 0.543 |  | 0.697 | 1.540 |  | 0.172 | 1.650 | 0.917 |
|  | K vs. E | 0.615 | 0.546 |  | -0.174 | 1.527 |  | 0.789 | 1.635 | 0.629 |
|  | K vs. F | 0.585 | 0.425 |  | 0.610 | 95.519 |  | -0.025 | 95.520 | 1.000 |
|  | K vs. I | 0.461 | 0.497 |  | 0.830 | 116.600 |  | -0.370 | 116.601 | 0.997 |
|  | K vs. J | 0.557 | 0.733 |  | 0.327 | 1.918 |  | 0.231 | 2.059 | 0.911 |
|  | K vs. H | 0.459 | 0.734 |  | 0.229 | 1.918 |  | 0.231 | 2.059 | 0.911 |
|  | G vs. E | -0.399 | 0.767 |  | -0.232 | 0.890 |  | -0.167 | 1.175 | 0.887 |
|  | G vs. F | -0.269 | 0.526 |  | -0.202 | 1.528 |  | -0.067 | 1.620 | 0.967 |
|  | E vs. F | 0.089 | 0.531 |  | -0.125 | 1.529 |  | 0.213 | 1.617 | 0.895 |
|  | I vs. J | 0.038 | 0.725 |  | 0.268 | 1.926 |  | -0.231 | 2.059 | 0.911 |
|  | I vs. H | -0.060 | 0.727 |  | 0.171 | 1.927 |  | -0.231 | 2.059 | 0.911 |
|  | J vs. H | - | - |  | - | - |  | - | - | - |
| Sustained pain relief over 24 hours | K vs. D | - | - |  | - | - |  | - | - | - |
|  | K vs. G | 0.569 | 0.095 |  | 0.632 | 0.254 |  | -0.062 | 0.283 | 0.826 |
|  | K vs. E | 0.410 | 0.099 |  | 0.480 | 0.254 |  | -0.070 | 0.282 | 0.803 |
|  | K vs. F | - | - |  | - | - |  | - | - | - |
|  | K vs. I | - | - |  | - | - |  | - | - | - |
|  | K vs. J | 0.222 | 0.087 |  | 0.290 | 0.208 |  | -0.068 | 0.232 | 0.770 |
|  | K vs. H | 0.203 | 0.089 |  | 0.270 | 0.209 |  | -0.068 | 0.232 | 0.770 |
|  | G vs. E | -0.141 | 0.161 |  | -0.168 | 0.119 |  | 0.027 | 0.200 | 0.893 |
|  | G vs. F | -0.036 | 0.077 |  | -0.182 | 0.239 |  | -0.018 | 0.252 | 0.943 |
|  | E vs. F | 0.126 | 0.081 |  | 0.108 | 0.238 |  | 0.017 | 0.248 | 0.944 |
|  | I vs. J | 0.048 | 0.081 |  | -0.020 | 0.216 |  | 0.068 | 0.232 | 0.770 |
|  | I vs. H | 0.028 | 0.082 |  | -0.040 | 0.216 |  | 0.068 | 0.232 | 0.770 |
|  | J vs. H | - | - |  | - | - |  | - | - | - |
| Freedom from photophobia at 2 hours | B vs. C | 0.025 | 0.029 |  | 0.102 | 28.216 |  | -0.077 | 28.216 | 0.998 |
|  | B vs. A | -0.076 | 0.041 |  | -0.134 | 0.100 |  | 0.058 | 0.106 | 0.588 |
|  | B vs. K | -0.237 | 0.035 |  | -0.199 | 14.003 |  | -0.038 | 14.003 | 0.998 |
|  | C vs. A | -0.112 | 0.040 |  | -0.086 | 0.100 |  | -0.027 | 0.106 | 0.801 |
|  | C vs. K | -0.262 | 0.034 |  | -0.186 | 27.992 |  | -0.077 | 27.992 | 0.998 |
|  | A vs. K | -0.146 | 0.048 |  | -0.189 | 0.113 |  | 0.043 | 0.127 | 0.732 |
|  | K vs. D | 0.418 | 0.065 |  | 0.502 | 57.859 |  | -0.084 | 57.859 | 0.999 |
|  | K vs. G | 0.425 | 0.076 |  | 0.227 | 0.100 |  | 0.198 | 0.125 | 0.113 |
|  | K vs. E | 0.128 | 0.079 |  | 0.154 | 0.201 |  | -0.026 | 0.218 | 0.904 |
|  | K vs. F | 0.254 | 0.058 |  | 0.507 | 0.171 |  | -0.253 | 0.176 | 0.152 |
|  | K vs. I | 0.022 | 0.067 |  | 0.414 | 62.512 |  | -0.193 | 62.512 | 0.998 |
|  | K vs. J | 0.218 | 0.105 |  | 0.430 | 0.248 |  | -0.212 | 0.276 | 0.442 |
|  | K vs. H | 0.140 | 0.109 |  | 0.352 | 0.250 |  | -0.212 | 0.276 | 0.442 |
|  | G vs. E | -0.340 | 0.148 |  | -0.184 | 0.085 |  | -0.155 | 0.169 | 0.358 |
|  | G vs. F | -0.065 | 0.046 |  | -0.290 | 0.195 |  | 0.225 | 0.202 | 0.266 |
|  | E vs. F | 0.122 | 0.071 |  | 0.299 | 0.177 |  | -0.177 | 0.187 | 0.342 |
|  | I vs. J | 0.062 | 0.100 |  | -0.150 | 0.254 |  | 0.212 | 0.276 | 0.442 |
|  | I vs. H | -0.016 | 0.104 |  | -0.228 | 0.256 |  | 0.212 | 0.276 | 0.442 |
|  | J vs. H | - | - |  | - | - |  | - | - | - |
| Freedom from phonophobia at 2 hours | B vs. C | 0.016 | 0.093 |  | -0.018 | 24.492 |  | 0.034 | 24.492 | 0.999 |
|  | B vs. A | -0.044 | 0.145 |  | 0.014 | 0.327 |  | -0.058 | 0.357 | 0.870 |
|  | B vs. K | -0.138 | 0.094 |  | -0.155 | 12.235 |  | 0.017 | 12.236 | 0.999 |
|  | C vs. A | -0.059 | 0.145 |  | -0.009 | 0.327 |  | -0.050 | 0.357 | 0.888 |
|  | C vs. K | -0.154 | 0.094 |  | -0.188 | 24.508 |  | 0.034 | 24.508 | 0.999 |
|  | A vs. K | -0.122 | 0.145 |  | -0.011 | 0.325 |  | -0.110 | 0.357 | 0.757 |
|  | K vs. D | 0.410 | 0.101 |  | 0.331 | 55.039 |  | 0.078 | 55.039 | 0.999 |
|  | K vs. G | 0.217 | 0.078 |  | -0.289 | 0.125 |  | 0.507 | 0.148 | **0.001** |
|  | K vs. E | 0.192 | 0.122 |  | 0.152 | 0.330 |  | 0.040 | 0.356 | 0.911 |
|  | K vs. F | 0.191 | 0.044 |  | 1.108 | 0.169 |  | -0.916 | 0.172 | **0.000** |
|  | K vs. I | 0.248 | 0.115 |  | 0.208 | 55.641 |  | 0.041 | 55.642 | 0.999 |
|  | K vs. J | 0.241 | 0.175 |  | 0.148 | 0.444 |  | 0.093 | 0.481 | 0.846 |
|  | K vs. H | 0.261 | 0.175 |  | 0.168 | 0.444 |  | 0.093 | 0.481 | 0.846 |
|  | G vs. E | -0.101 | 0.168 |  | 0.220 | 0.147 |  | -0.321 | 0.223 | 0.151 |
|  | G vs. F | 0.175 | 0.089 |  | -0.111 | 0.313 |  | 0.286 | 0.326 | 0.380 |
|  | E vs. F | 0.012 | 0.103 |  | 0.458 | 0.255 |  | -0.446 | 0.275 | 0.105 |
|  | I vs. J | -0.032 | 0.170 |  | 0.061 | 0.450 |  | -0.093 | 0.481 | 0.846 |
|  | I vs. H | -0.012 | 0.169 |  | 0.081 | 0.450 |  | -0.093 | 0.481 | 0.846 |
|  | J vs. H | - | - |  | - | - |  | - | - | - |

A: lasmiditan 50 mg; B: lasmiditan 100 mg; C: lasmiditan 200 mg; D: rimegepant 75 mg; E: ubrogepant 25 mg; F: ubrogepant 50 mg; G: ubrogepant 100 mg; H: zavegepant 5 mg; I: zavegepant 10 mg; J: zavegepant 20 mg; K: placebo; MBS: most bothersome sympto

# Table S6: SUCRA values for all outcomes

|  | **Pain freedom at 2 hours** | **Pain relief at 2 hours** | **MBS freedom at 2 hours** | **Sustained pain freedom over 24 hours** | **Sustained pain relief over 24 hours** | **Freedom from photophobia at 2 hours** | **Freedom from phonophobia at 2 hours** |
| --- | --- | --- | --- | --- | --- | --- | --- |
| Treatment | SUCRA | SUCRA | SUCRA | SUCRA | SUCRA | SUCRA | SUCRA |
| Lasmiditan 200 mg | 0.7371 | 0.9124 | 0.4644 | 0.6543 | NA | 0.6388 | 0.4385 |
| Lasmiditan 100 mg | 0.4294 | 0.9375 | 0.3870 | 0.5420 | NA | 0.5425 | 0.3984 |
| Lasmiditan 50 mg | 0.2308 | 0.5837 | 0.2334 | 0.4150 | NAN | 0.2943 | 0.3265 |
| Rimegepant 75 mg | 0.6862 | 0.7955 | 0.7876 | 0.3005 | 0.6307 | 0.9560 | 0.9027 |
| Ubrogepant 100 mg | 0.7879 | 0.5466 | 0.7084 | 0.7367 | 0.7611 | 0.7200 | 0.3147 |
| Ubrogepant 50 mg | 0.5735 | 0.5489 | 0.7858 | 0.5895 | 0.6998 | 0.6251 | 0.6899 |
| Ubrogepant 25 mg | 0.5315 | 0.4523 | 0.5047 | 0.5441 | 0.4636 | 0.2164 | 0.5105 |
| Zavegepant 20 mg | NA | 0.2874 | NA | 0.5423 | 1.0000 | 0.5918 | 0.5793 |
| Zavegepant 10 mg | 0.5056 | 0.2793 | 0.5751 | 0.5098 | 0.2072 | 0.4892 | 0.6382 |
| Zavegepant 5 mg | NA | 0.1479 | NA | 0.4897 | 0.2343 | 0.3816 | 0.6200 |
| Placebo | 0.0181 | 0.0085 | 0.0534 | 0.1761 | 0.0032 | 0.0442 | 0.0814 |

SUCRA values range from 0 to 1, where 1 reflects the best treatment with no uncertainty and 0 reflects the worst treatment with no uncertainty; higher value has the greatest probability of being the best. NA: not available; SUCRA: surface under the cumulative ranking curve.

# Table S7: CINeMA ratings for all comparisons of the seven outcomes

| **Comparison** | **Nature of evidence** | **Confidence level** | **Downgrading** |
| --- | --- | --- | --- |
| **Pain freedom at 2 hours** | | | |
| Lasmiditan 100 mg vs.  Lasmiditan 200 mg | mixed | Very low | Study limitations, Imprecisions, Heterogeneity |
| Lasmiditan 100 mg vs.  Lasmiditan 50 mg | mixed | Low | Serious Imprecisions |
| Lasmiditan 100 mg vs.  Placebo | mixed | Low | Imprecisions, Heterogeneity |
| Lasmiditan 200 mg vs.  Lasmiditan 50 mg | mixed | Moderate | Heterogeneity |
| Lasmiditan 200 mg vs.  Placebo | mixed | Low | Serious Imprecisions |
| Lasmiditan 50 mg vs.  Placebo | mixed | Low | Serious Imprecisions |
| Rimegepant 75 mg vs.  Placebo | mixed | High |  |
| Ubrogepant 100 mg vs.  Placebo | mixed | High |  |
| Ubrogepant 25 mg vs.  Placebo | mixed | Moderate | Heterogeneity |
| Ubrogepant 50 mg vs.  Placebo | mixed | Moderate | Heterogeneity |
| Zavegepant 10 mg vs.  Placebo | mixed | Low | Serious Imprecisions |
| Ubrogepant 100 mg vs.  Ubrogepant 25 mg | mixed | Low | Serious Imprecisions |
| Ubrogepant 100 mg vs.  Ubrogepant 50 mg | mixed | Very low | Study limitations, Serious Imprecisions |
| Ubrogepant 25 mg vs.  Ubrogepant 50 mg | mixed | Low | Imprecisions, Heterogeneity |
| Lasmiditan 100 mg vs.  Rimegepant 75 mg | indirect | Moderate | Imprecisions |
| Lasmiditan 100 mg vs.  Ubrogepant 100 mg | indirect | Low | Imprecisions, Heterogeneity |
| Lasmiditan 100 mg vs.  Ubrogepant 25 mg | indirect | Moderate | Imprecisions |
| Lasmiditan 100 mg vs.  Ubrogepant 50 mg | indirect | Moderate | Imprecisions |
| Lasmiditan 100 mg vs.  Zavegepant 10 mg | indirect | Very low | Serious Imprecisions, Heterogeneity |
| Lasmiditan 200 mg vs.  Rimegepant 75 mg | indirect | Moderate | Imprecisions |
| Lasmiditan 200 mg vs.  Ubrogepant 100 mg | indirect | Moderate | Imprecisions |
| Lasmiditan 200 mg vs.  Ubrogepant 25 mg | indirect | Low | Serious Imprecisions |
| Lasmiditan 200 mg vs.  Ubrogepant 50 mg | indirect | Very low | Serious Imprecisions, Heterogeneity |
| Lasmiditan 200 mg vs.  Zavegepant 10 mg | indirect | Low | Serious Imprecisions |
| Lasmiditan 50 mg vs.  Rimegepant 75 mg | indirect | Moderate | Imprecisions |
| Lasmiditan 50 mg vs.  Ubrogepant 100 mg | indirect | Moderate | Imprecisions |
| Lasmiditan 50 mg vs.  Ubrogepant 25 mg | indirect | Moderate | Imprecisions |
| Lasmiditan 50 mg vs.  Ubrogepant 50 mg | indirect | Low | Serious Imprecisions |
| Lasmiditan 50 mg vs.  Zavegepant 10 mg | indirect | Low | Imprecisions, Heterogeneity |
| Rimegepant 75 mg vs.  Ubrogepant 100 mg | indirect | Low | Serious Imprecisions |
| Rimegepant 75 mg vs.  Ubrogepant 25 mg | indirect | Very low | Serious Imprecisions, Heterogeneity |
| Rimegepant 75 mg vs.  Ubrogepant 50 mg | indirect | Moderate | Imprecisions |
| Rimegepant 75 mg vs.  Zavegepant 10 mg | indirect | Low | Serious Imprecisions |
| Ubrogepant 100 mg vs.  Zavegepant 10 mg | indirect | Low | Serious Imprecisions |
| Ubrogepant 25 mg vs.  Zavegepant 10 mg | indirect | Moderate | Imprecisions |
| Ubrogepant 50 mg vs.  Zavegepant 10 mg | indirect | Moderate | Imprecisions |
| **Pain relief at 2 hours** | | | |
| Lasmiditan 100 mg vs.  Lasmiditan 200 mg | mixed | Moderate | Imprecisions |
| Lasmiditan 100 mg vs.  Lasmiditan 50 mg | mixed | High |  |
| Lasmiditan 100 mg vs.  Placebo | mixed | Low | Imprecisions, Heterogeneity |
| Lasmiditan 200 mg vs.  Lasmiditan 50 mg | mixed | High |  |
| Lasmiditan 200 mg vs.  Placebo | mixed | Low | Imprecisions, Heterogeneity |
| Lasmiditan 50 mg vs.  Placebo | mixed | High |  |
| Rimegepant 75 mg vs.  Placebo | mixed | Moderate | Heterogeneity |
| Ubrogepant 100 mg vs.  Placebo | mixed | High |  |
| Ubrogepant 25 mg vs.  Placebo | mixed | Moderate | Heterogeneity |
| Ubrogepant 50 mg vs.  Placebo | mixed | Moderate | Heterogeneity |
| Zavegepant 10 mg vs.  Placebo | mixed | High |  |
| Zavegepant 20 mg vs.  Placebo | mixed | High |  |
| Zavegepant 5 mg vs.  Placebo | mixed | Moderate | Imprecisions |
| Ubrogepant 100 mg vs.  Ubrogepant 50 mg | mixed | Moderate | Imprecisions |
| Ubrogepant 25 mg vs.  Ubrogepant 50 mg | mixed | Moderate | Imprecisions |
| Zavegepant 10 mg vs.  Zavegepant 20 mg | mixed | Moderate | Imprecisions |
| Zavegepant 10 mg vs.  Zavegepant 5 mg | mixed | Moderate | Imprecisions |
| Zavegepant 20 mg vs.  Zavegepant 5 mg | mixed | Low | Serious Imprecisions |
| Lasmiditan 100 mg vs.  Rimegepant 75 mg | indirect | Low | Serious Imprecisions |
| Lasmiditan 100 mg vs.  Ubrogepant 100 mg | indirect | Low | Serious Imprecisions |
| Lasmiditan 100 mg vs.  Ubrogepant 25 mg | indirect | High |  |
| Lasmiditan 100 mg vs.  Ubrogepant 50 mg | indirect | High |  |
| Lasmiditan 100 mg vs.  Zavegepant 10 mg | indirect | High |  |
| Lasmiditan 100 mg vs.  Zavegepant 20 mg | indirect | High |  |
| Lasmiditan 100 mg vs.  Zavegepant 5 mg | indirect | High |  |
| Lasmiditan 200 mg vs.  Rimegepant 75 mg | indirect | Low | Serious Imprecisions |
| Lasmiditan 200 mg vs.  Ubrogepant 100 mg | indirect | Low | Serious Imprecisions |
| Lasmiditan 200 mg vs.  Ubrogepant 25 mg | indirect | High |  |
| Lasmiditan 200 mg vs.  Ubrogepant 50 mg | indirect | Moderate | Heterogeneity |
| Lasmiditan 200 mg vs.  Zavegepant 10 mg | indirect | High |  |
| Lasmiditan 200 mg vs.  Zavegepant 20 mg | indirect | High |  |
| Lasmiditan 200 mg vs.  Zavegepant 5 mg | indirect | High |  |
| Lasmiditan 50 mg vs.  Rimegepant 75 mg | indirect | Low | Serious Imprecisions |
| Lasmiditan 50 mg vs.  Ubrogepant 100 mg | indirect | Low | Serious Imprecisions |
| Lasmiditan 50 mg vs.  Ubrogepant 25 mg | indirect | Low | Imprecisions, Heterogeneity |
| Lasmiditan 50 mg vs.  Ubrogepant 50 mg | indirect | Low | Serious Imprecisions |
| Lasmiditan 50 mg vs.  Zavegepant 10 mg | indirect | Low | Imprecisions, Heterogeneity |
| Lasmiditan 50 mg vs.  Zavegepant 20 mg | indirect | Low | Serious Imprecisions |
| Lasmiditan 50 mg vs.  Zavegepant 5 mg | indirect | High |  |
| Rimegepant 75 mg vs.  Ubrogepant 100 mg | indirect | Low | Serious Imprecisions |
| Rimegepant 75 mg vs.  Ubrogepant 25 mg | indirect | Very low | Serious Imprecisions, Heterogeneity |
| Rimegepant 75 mg vs.  Ubrogepant 50 mg | indirect | Low | Serious Imprecisions |
| Rimegepant 75 mg vs.  Zavegepant 10 mg | indirect | High |  |
| Rimegepant 75 mg vs.  Zavegepant 20 mg | indirect | High |  |
| Rimegepant 75 mg vs.  Zavegepant 5 mg | indirect | High |  |
| Ubrogepant 100 mg vs.  Ubrogepant 25 mg | indirect | Low | Imprecisions, Heterogeneity |
| Ubrogepant 100 mg vs.  Zavegepant 10 mg | indirect | Very low | Serious Imprecisions, Heterogeneity |
| Ubrogepant 100 mg vs.  Zavegepant 20 mg | indirect | Low | Serious Imprecisions |
| Ubrogepant 100 mg vs.  Zavegepant 5 mg | indirect | Low | Serious Imprecisions |
| Ubrogepant 25 mg vs.  Zavegepant 10 mg | indirect | Very low | Serious Imprecisions, Heterogeneity |
| Ubrogepant 25 mg vs.  Zavegepant 20 mg | indirect | Low | Serious Imprecisions |
| Ubrogepant 25 mg vs.  Zavegepant 5 mg | indirect | Low | Serious Imprecisions |
| Ubrogepant 50 mg vs.  Zavegepant 10 mg | indirect | Low | Imprecisions, Heterogeneity |
| Ubrogepant 50 mg vs. Zavegepant 20 mg | indirect | Low | Serious Imprecisions |
| Ubrogepant 50 mg vs.  Zavegepant 5 mg | indirect | Moderate | Heterogeneity |
| **MBS freedom at 2 hours** | | | |
| Lasmiditan 100 mg vs.  Lasmiditan 200 mg | mixed | Low | Serious Imprecisions |
| Lasmiditan 100 mg vs.  Lasmiditan 50 mg | mixed | Low | Serious Imprecisions |
| Lasmiditan 100 mg vs.  Placebo | mixed | Low | Imprecisions, Heterogeneity |
| Lasmiditan 200 mg vs.  Lasmiditan 50 mg | mixed | Low | Serious Imprecisions |
| Lasmiditan 200 mg vs.  Placebo | mixed | Low | Imprecisions, Heterogeneity |
| Lasmiditan 50 mg vs.  Placebo | mixed | Low | Serious Imprecisions |
| Rimegepant 75 mg vs.  Placebo | mixed | Moderate | Heterogeneity |
| Ubrogepant 100 mg vs.  Placebo | mixed | Moderate | Heterogeneity |
| Ubrogepant 25 mg vs.  Placebo | mixed | Low | Serious Imprecisions |
| Ubrogepant 50 mg vs.  Placebo | mixed | Moderate | Heterogeneity |
| Zavegepant 10 mg vs.  Placebo | mixed | Low | Serious Imprecisions |
| Ubrogepant 100 mg vs.  Ubrogepant 50 mg | mixed | Low | Serious Imprecisions |
| Ubrogepant 25 mg vs.  Ubrogepant 50 mg | mixed | Very low | Serious Imprecisions, Heterogeneity |
| Lasmiditan 100 mg vs.  Rimegepant 75 mg | indirect | Very low | Serious Imprecisions, Heterogeneity |
| Lasmiditan 100 mg vs.  Ubrogepant 100 mg | indirect | Low | Serious Imprecisions |
| Lasmiditan 100 mg vs.  Ubrogepant 25 mg | indirect | Low | Imprecisions, Heterogeneity |
| Lasmiditan 100 mg vs.  Ubrogepant 50 mg | indirect | Low | Serious Imprecisions |
| Lasmiditan 100 mg vs.  Zavegepant 10 mg | indirect | Low | Serious Imprecisions |
| Lasmiditan 200 mg vs.  Rimegepant 75 mg | indirect | Low | Imprecisions, Heterogeneity |
| Lasmiditan 200 mg vs.  Ubrogepant 100 mg | indirect | Low | Serious Imprecisions |
| Lasmiditan 200 mg vs.  Ubrogepant 25 mg | indirect | Low | Imprecisions, Heterogeneity |
| Lasmiditan 200 mg vs.  Ubrogepant 50 mg | indirect | Low | Serious Imprecisions |
| Lasmiditan 200 mg vs.  Zavegepant 10 mg | indirect | Low | Serious Imprecisions |
| Lasmiditan 50 mg vs.  Rimegepant 75 mg | indirect | Low | Serious Imprecisions |
| Lasmiditan 50 mg vs.  Ubrogepant 100 mg | indirect | Low | Imprecisions, Heterogeneity |
| Lasmiditan 50 mg vs.  Ubrogepant 25 mg | indirect | Low | Serious Imprecisions |
| Lasmiditan 50 mg vs.  Ubrogepant 50 mg | indirect | Low | Imprecisions, Heterogeneity |
| Lasmiditan 50 mg vs.  Zavegepant 10 mg | indirect | Very low | Serious Imprecisions, Heterogeneity |
| Rimegepant 75 mg vs.  Ubrogepant 100 mg | indirect | Low | Serious Imprecisions |
| Rimegepant 75 mg vs.  Ubrogepant 25 mg | indirect | Very low | Serious Imprecisions, Heterogeneity |
| Rimegepant 75 mg vs.  Ubrogepant 50 mg | indirect | Low | Imprecisions, Heterogeneity |
| Rimegepant 75 mg vs.  Zavegepant 10 mg | indirect | Very low | Serious Imprecisions, Heterogeneity |
| Ubrogepant 100 mg vs.  Ubrogepant 25 mg | indirect | Low | Imprecisions, Heterogeneity |
| Ubrogepant 100 mg vs.  Zavegepant 10 mg | indirect | Low | Serious Imprecisions |
| Ubrogepant 25 mg vs.  Zavegepant 10 mg | indirect | Low | Imprecisions, Heterogeneity |
| Ubrogepant 50 mg vs.  Zavegepant 10 mg | indirect | Low | Serious Imprecisions |
| **Sustained pain freedom over 24 hours** | | | |
| Lasmiditan 100 mg vs.  Lasmiditan 200 mg | mixed | Moderate | Imprecisions |
| Lasmiditan 100 mg vs.  Lasmiditan 50 mg | mixed | Low | Serious Imprecisions |
| Lasmiditan 100 mg vs.  Placebo | mixed | Low | Serious Imprecisions |
| Lasmiditan 200 mg vs.  Lasmiditan 50 mg | mixed | Low | Serious Imprecisions |
| Lasmiditan 200 mg vs.  Placebo | mixed | Low | Serious Imprecisions |
| Lasmiditan 50 mg vs.  Placebo | mixed | Low | Serious Imprecisions |
| Rimegepant 75 mg vs.  Placebo | mixed | Low | Imprecisions, Heterogeneity |
| Ubrogepant 100 mg vs.  Placebo | mixed | Low | Imprecisions, Heterogeneity |
| Ubrogepant 25 mg vs.  Placebo | mixed | Low | Serious Imprecisions |
| Ubrogepant 50 mg vs.  Placebo | mixed | Moderate | Imprecisions |
| Zavegepant 10 mg vs.  Placebo | mixed | Low | Serious Imprecisions |
| Zavegepant 20 mg vs.  Placebo | mixed | Low | Serious Imprecisions |
| Zavegepant 5 mg vs.  Placebo | mixed | Moderate | Imprecisions |
| Ubrogepant 100 mg vs.  Ubrogepant 25 mg | mixed | Low | Serious Imprecisions |
| Ubrogepant 100 mg vs.  Ubrogepant 50 mg | mixed | Low | Serious Imprecisions |
| Ubrogepant 25 mg vs.  Ubrogepant 50 mg | mixed | Low | Serious Imprecisions |
| Zavegepant 10 mg vs.  Zavegepant 20 mg | mixed | Low | Serious Imprecisions |
| Zavegepant 10 mg vs.  Zavegepant 5 mg | mixed | Low | Serious Imprecisions |
| Zavegepant 20 mg vs.  Zavegepant 5 mg | mixed | Very low | Serious Imprecisions, Heterogeneity |
| Lasmiditan 100 mg vs.  Rimegepant 75 mg | indirect | Very low | Serious Imprecisions, Heterogeneity |
| Lasmiditan 100 mg vs.  Ubrogepant 100 mg | indirect | Low | Serious Imprecisions |
| Lasmiditan 100 mg vs.  Ubrogepant 25 mg | indirect | Low | Serious Imprecisions |
| Lasmiditan 100 mg vs.  Ubrogepant 50 mg | indirect | Low | Imprecisions, Heterogeneity |
| Lasmiditan 100 mg vs.  Zavegepant 10 mg | indirect | Low | Imprecisions, Heterogeneity |
| Lasmiditan 100 mg vs.  Zavegepant 20 mg | indirect | Low | Serious Imprecisions |
| Lasmiditan 100 mg vs.  Zavegepant 5 mg | indirect | Low | Serious Imprecisions |
| Lasmiditan 200 mg vs.  Rimegepant 75 mg | indirect | Low | Imprecisions, Heterogeneity |
| Lasmiditan 200 mg vs.  Ubrogepant 100 mg | indirect | Very low | Serious Imprecisions, Heterogeneity |
| Lasmiditan 200 mg vs.  Ubrogepant 25 mg | indirect | Low | Serious Imprecisions |
| Lasmiditan 200 mg vs.  Ubrogepant 50 mg | indirect | Low | Serious Imprecisions |
| Lasmiditan 200 mg vs.  Zavegepant 10 mg | indirect | Low | Serious Imprecisions |
| Lasmiditan 200 mg vs.  Zavegepant 20 mg | indirect | Low | Serious Imprecisions |
| Lasmiditan 200 mg vs.  Zavegepant 5 mg | indirect | Very low | Serious Imprecisions, Heterogeneity |
| Lasmiditan 50 mg vs.  Rimegepant 75 mg | indirect | Low | Serious Imprecisions |
| Lasmiditan 50 mg vs.  Ubrogepant 100 mg | indirect | Low | Serious Imprecisions |
| Lasmiditan 50 mg vs.  Ubrogepant 25 mg | indirect | Moderate | Imprecisions |
| Lasmiditan 50 mg vs.  Ubrogepant 50 mg | indirect | Low | Serious Imprecisions |
| Lasmiditan 50 mg vs.  Zavegepant 10 mg | indirect | Low | Serious Imprecisions |
| Lasmiditan 50 mg vs.  Zavegepant 20 mg | indirect | Moderate | Imprecisions |
| Lasmiditan 50 mg vs.  Zavegepant 5 mg | indirect | Low | Serious Imprecisions |
| Rimegepant 75 mg vs.  Ubrogepant 100 mg | indirect | Low | Serious Imprecisions |
| Rimegepant 75 mg vs.  Ubrogepant 25 mg | indirect | Moderate | Imprecisions |
| Rimegepant 75 mg vs.  Ubrogepant 50 mg | indirect | Moderate | Imprecisions |
| Rimegepant 75 mg vs.  Zavegepant 10 mg | indirect | Low | Serious Imprecisions |
| Rimegepant 75 mg vs.  Zavegepant 20 mg | indirect | Moderate | Imprecisions |
| Rimegepant 75 mg vs.  Zavegepant 5 mg | indirect | Very low | Serious Imprecisions, Heterogeneity |
| Ubrogepant 100 mg vs.  Zavegepant 10 mg | indirect | Very low | Serious Imprecisions, Heterogeneity |
| Ubrogepant 100 mg vs.  Zavegepant 20 mg | indirect | Low | Serious Imprecisions |
| Ubrogepant 100 mg vs.  Zavegepant 5 mg | indirect | Low | Serious Imprecisions |
| Ubrogepant 25 mg vs.  Zavegepant 10 mg | indirect | Very low | Serious Imprecisions, Heterogeneity |
| Ubrogepant 25 mg vs.  Zavegepant 20 mg | indirect | Low | Serious Imprecisions |
| Ubrogepant 25 mg vs.  Zavegepant 5 mg | indirect | Very low | Serious Imprecisions, Heterogeneity |
| Ubrogepant 50 mg vs.  Zavegepant 10 mg | indirect | Low | Serious Imprecisions |
| Ubrogepant 50 mg vs.  Zavegepant 20 mg | indirect | Very low | Serious Imprecisions, Heterogeneity |
| Ubrogepant 50 mg vs.  Zavegepant 5 mg | indirect | Low | Serious Imprecisions |
| **Sustained pain relief over 24 hours** | | | |
| Rimegepant 75 mg vs.  Placebo | mixed | High |  |
| Ubrogepant 100 mg vs.  Placebo | mixed | Moderate | Heterogeneity |
| Ubrogepant 25 mg vs.  Placebo | mixed | High |  |
| Ubrogepant 50 mg vs.  Placebo | mixed | High |  |
| Zavegepant 10 mg vs.  Placebo | mixed | Low | Imprecisions, Heterogeneity |
| Zavegepant 20 mg vs.  Placebo | mixed | Low | Imprecisions, Heterogeneity |
| Zavegepant 5 mg vs.  Placebo | mixed | Low | Imprecisions, Heterogeneity |
| Ubrogepant 100 mg vs.  Ubrogepant 25 mg | mixed | Very low | Serious Imprecisions, Heterogeneity |
| Ubrogepant 100 mg vs.  Ubrogepant 50 mg | mixed | Low | Serious Imprecisions |
| Ubrogepant 25 mg vs.  Ubrogepant 50 mg | mixed | Low | Serious Imprecisions |
| Zavegepant 10 mg vs.  Zavegepant 20 mg | mixed | Low | Serious Imprecisions |
| Zavegepant 10 mg vs.  Zavegepant 5 mg | mixed | Low | Serious Imprecisions |
| Zavegepant 20 mg vs.  Zavegepant 5 mg | mixed | Low | Serious Imprecisions |
| Rimegepant 75 mg vs.  Ubrogepant 100 mg | indirect | Very low | Serious Imprecisions, Heterogeneity |
| Rimegepant 75 mg vs.  Ubrogepant 25 mg | indirect | Low | Serious Imprecisions |
| Rimegepant 75 mg vs.  Ubrogepant 50 mg | indirect | Very low | Serious Imprecisions, Heterogeneity |
| Rimegepant 75 mg vs.  Zavegepant 10 mg | indirect | High |  |
| Rimegepant 75 mg vs.  Zavegepant 20 mg | indirect | Moderate | Heterogeneity |
| Rimegepant 75 mg vs.  Zavegepant 5 mg | indirect | High |  |
| Ubrogepant 100 mg vs.  Zavegepant 10 mg | indirect | High |  |
| Ubrogepant 100 mg vs.  Zavegepant 20 mg | indirect | Moderate |  |
| Ubrogepant 100 mg vs.  Zavegepant 5 mg | indirect | High |  |
| Ubrogepant 25 mg vs.  Zavegepant 10 mg | indirect | Moderate | Heterogeneity |
| Ubrogepant 25 mg vs.  Zavegepant 20 mg | indirect | Low | Serious Imprecisions |
| Ubrogepant 25 mg vs.  Zavegepant 5 mg | indirect | Low | Serious Imprecisions |
| Ubrogepant 50 mg vs.  Zavegepant 10 mg | indirect | High |  |
| Ubrogepant 50 mg vs.  Zavegepant 20 mg | indirect | High |  |
| Ubrogepant 50 mg vs.  Zavegepant 5 mg | indirect | High |  |
| **Freedom from photophobia at 2 hours** | | | |
| Lasmiditan 100 mg vs.  Lasmiditan 200 mg | mixed | Low | Serious Imprecisions |
| Lasmiditan 100 mg vs.  Lasmiditan 50 mg | mixed | Moderate | Heterogeneity |
| Lasmiditan 100 mg vs. Placebo | mixed | Moderate | Heterogeneity |
| Lasmiditan 200 mg vs.  Lasmiditan 50 mg | mixed | High |  |
| Lasmiditan 200 mg vs.  Placebo | mixed | Moderate | Heterogeneity |
| Lasmiditan 50 mg vs.  Placebo | mixed | Moderate | Heterogeneity |
| Rimegepant 75 mg vs.  Placebo | mixed | High |  |
| Ubrogepant 100 mg vs.  Placebo | mixed | Moderate | Heterogeneity |
| Ubrogepant 25 mg vs.  Placebo | mixed | Low | Serious Imprecisions |
| Ubrogepant 50 mg vs.  Placebo | mixed | Moderate | Heterogeneity |
| Zavegepant 10 mg vs.  Placebo | mixed | High |  |
| Zavegepant 20 mg vs.  Placebo | mixed | High |  |
| Zavegepant 5 mg vs.  Placebo | mixed | Low | Serious Imprecisions |
| Ubrogepant 100 mg vs.  Ubrogepant 25 mg | mixed | Moderate | Heterogeneity |
| Ubrogepant 100 mg vs.  Ubrogepant 50 mg | mixed | Very low | Study limitations, Serious Imprecisions |
| Ubrogepant 25 mg vs.  Ubrogepant 50 mg | mixed | Moderate | Heterogeneity |
| Zavegepant 10 mg vs.  Zavegepant 20 mg | mixed | Very low | Serious Imprecisions, Heterogeneity |
| Zavegepant 10 mg vs.  Zavegepant 5 mg | mixed | Low | Serious Imprecisions |
| Zavegepant 20 mg vs.  Zavegepant 5 mg | mixed | Low | Serious Imprecisions |
| Lasmiditan 100 mg vs.  Rimegepant 75 mg | indirect | Moderate | Heterogeneity |
| Lasmiditan 100 mg vs.  Ubrogepant 100 mg | indirect | Low | Serious Imprecisions |
| Lasmiditan 100 mg vs.  Ubrogepant 25 mg | indirect | Very low | Serious Imprecisions, Heterogeneity |
| Lasmiditan 100 mg vs.  Ubrogepant 50 mg | indirect | Low | Serious Imprecisions |
| Lasmiditan 100 mg vs.  Zavegepant 10 mg | indirect | Very low | Serious Imprecisions, Incoherence |
| Lasmiditan 100 mg vs.  Zavegepant 20 mg | indirect | Very low | Serious Imprecisions, Incoherence |
| Lasmiditan 100 mg vs.  Zavegepant 5 mg | indirect | Low | Serious Imprecisions |
| Lasmiditan 200 mg vs.  Rimegepant 75 mg | indirect | Moderate | Heterogeneity |
| Lasmiditan 200 mg vs.  Ubrogepant 100 mg | indirect | Low | Serious Imprecisions |
| Lasmiditan 200 mg vs.  Ubrogepant 25 mg | indirect | Low | Serious Imprecisions |
| Lasmiditan 200 mg vs.  Ubrogepant 50 mg | indirect | Low | Serious Imprecisions |
| Lasmiditan 200 mg vs.  Zavegepant 10 mg | indirect | Very low | Serious Imprecisions, Heterogeneity |
| Lasmiditan 200 mg vs.  Zavegepant 20 mg | indirect | Low | Serious Imprecisions |
| Lasmiditan 200 mg vs.  Zavegepant 5 mg | indirect | Low | Serious Imprecisions |
| Lasmiditan 50 mg vs.  Rimegepant 75 mg | indirect | High |  |
| Lasmiditan 50 mg vs.  Ubrogepant 100 mg | indirect | Moderate | Heterogeneity |
| Lasmiditan 50 mg vs.  Ubrogepant 25 mg | indirect | Very low | Serious Imprecisions, Incoherence |
| Lasmiditan 50 mg vs.  Ubrogepant 50 mg | indirect | Low | Serious Imprecisions |
| Lasmiditan 50 mg vs.  Zavegepant 10 mg | indirect | Low | Serious Imprecisions |
| Lasmiditan 50 mg vs.  Zavegepant 20 mg | indirect | Very low | Serious Imprecisions, Incoherence |
| Lasmiditan 50 mg vs.  Zavegepant 5 mg | indirect | Low | Serious Imprecisions |
| Rimegepant 75 mg vs.  Ubrogepant 100 mg | indirect | Low | Serious Imprecisions |
| Rimegepant 75 mg vs.  Ubrogepant 25 mg | indirect | Moderate | Heterogeneity |
| Rimegepant 75 mg vs.  Ubrogepant 50 mg | indirect | Low | Serious Imprecisions |
| Rimegepant 75 mg vs.  Zavegepant 10 mg | indirect | Moderate | Heterogeneity |
| Rimegepant 75 mg vs.  Zavegepant 20 mg | indirect | Low | Serious Imprecisions |
| Rimegepant 75 mg vs.  Zavegepant 5 mg | indirect | Moderate | Heterogeneity |
| Ubrogepant 100 mg vs.  Zavegepant 10 mg | indirect | Very low | Serious Imprecisions, Heterogeneity |
| Ubrogepant 100 mg vs.  Zavegepant 20 mg | indirect | Low | Serious Imprecisions |
| Ubrogepant 100 mg vs.  Zavegepant 5 mg | indirect | Very low | Serious Imprecisions, Incoherence |
| Ubrogepant 25 mg vs.  Zavegepant 10 mg | indirect | Low | Serious Imprecisions |
| Ubrogepant 25 mg vs.  Zavegepant 20 mg | indirect | Very low | Serious Imprecisions, Incoherence |
| Ubrogepant 25 mg vs.  Zavegepant 5 mg | indirect | Low | Serious Imprecisions |
| Ubrogepant 50 mg vs.  Zavegepant 10 mg | indirect | Low | Serious Imprecisions |
| Ubrogepant 50 mg vs.  Zavegepant 20 mg | indirect | Low | Serious Imprecisions |
| Ubrogepant 50 mg vs.  Zavegepant 5 mg | indirect | Low | Serious Imprecisions |
| **Freedom from phonophobia at 2 hours** | | | |
| Lasmiditan 100 mg vs.  Lasmiditan 200 mg | mixed | Very low | Serious Imprecisions, Heterogeneity |
| Lasmiditan 100 mg vs.  Lasmiditan 50 mg | mixed | Low | Serious Imprecisions |
| Lasmiditan 100 mg vs.  Placebo | mixed | Very low | Serious Imprecisions, Heterogeneity |
| Lasmiditan 200 mg vs.  Lasmiditan 50 mg | mixed | High |  |
| Lasmiditan 200 mg vs.  Placebo | mixed | Very low | Serious Imprecisions, Incoherence |
| Lasmiditan 50 mg vs.  Placebo | mixed | Low | Serious Imprecisions |
| Rimegepant 75 mg vs.  Placebo | mixed | Moderate | Incoherence |
| Ubrogepant 100 mg vs.  Placebo | mixed | Very low | Serious Imprecisions, Serious Incoherence |
| Ubrogepant 25 mg vs.  Placebo | mixed | Moderate | Heterogeneity |
| Ubrogepant 50 mg vs.  Placebo | mixed | Moderate | Heterogeneity |
| Zavegepant 10 mg vs.  Placebo | mixed | Low | Heterogeneity, Incoherence |
| Zavegepant 20 mg vs.  Placebo | mixed | Low | Serious Imprecisions |
| Zavegepant 5 mg vs.  Placebo | mixed | Low | Serious Imprecisions |
| Ubrogepant 100 mg vs.  Ubrogepant 25 mg | mixed | Very low | Serious Imprecisions, Heterogeneity |
| Ubrogepant 100 mg vs.  Ubrogepant 50 mg | mixed | Low | Study limitations, Heterogeneity |
| Ubrogepant 25 mg vs.  Ubrogepant 50 mg | mixed | Low | Serious Imprecisions |
| Zavegepant 10 mg vs.  Zavegepant 20 mg | mixed | Low | Serious Imprecisions |
| Zavegepant 10 mg vs.  Zavegepant 5 mg | mixed | Low | Serious Imprecisions |
| Zavegepant 20 mg vs.  Zavegepant 5 mg | mixed | Very low | Serious Imprecisions, Incoherence |
| Lasmiditan 100 mg vs.  Rimegepant 75 mg | indirect | Low | Heterogeneity, Incoherence |
| Lasmiditan 100 mg vs.  Ubrogepant 100 mg | indirect | Very low | Serious Imprecisions, Heterogeneity |
| Lasmiditan 100 mg vs.  Ubrogepant 25 mg | indirect | Very low | Serious Imprecisions, Incoherence |
| Lasmiditan 100 mg vs.  Ubrogepant 50 mg | indirect | Very low | Serious Imprecisions, Heterogeneity, Incoherence |
| Lasmiditan 100 mg vs.  Zavegepant 10 mg | indirect | Very low | Serious Imprecisions, Incoherence |
| Lasmiditan 100 mg vs.  Zavegepant 20 mg | indirect | Very low | Serious Imprecisions, Heterogeneity, Incoherence |
| Lasmiditan 100 mg vs.  Zavegepant 5 mg | indirect | Very low | Serious Imprecisions, Incoherence |
| Lasmiditan 200 mg vs.  Rimegepant 75 mg | indirect | Low | Heterogeneity, Incoherence |
| Lasmiditan 200 mg vs.  Ubrogepant 100 mg | indirect | Very low | Serious Imprecisions, Heterogeneity |
| Lasmiditan 200 mg vs.  Ubrogepant 25 mg | indirect | Very low | Serious Imprecisions, Heterogeneity |
| Lasmiditan 200 mg vs.  Ubrogepant 50 mg | indirect | Very low | Serious Imprecisions, Heterogeneity |
| Lasmiditan 200 mg vs.  Zavegepant 10 mg | indirect | Very low | Serious Imprecisions, Incoherence |
| Lasmiditan 200 mg vs.  Zavegepant 20 mg | indirect | Very low | Serious Imprecisions, Heterogeneity |
| Lasmiditan 200 mg vs.  Zavegepant 5 mg | indirect | Very low | Serious Imprecisions, Incoherence |
| Lasmiditan 50 mg vs.  Rimegepant 75 mg | indirect | Low | Heterogeneity, Incoherence |
| Lasmiditan 50 mg vs.  Ubrogepant 100 mg | indirect | Very low | Serious Imprecisions, Incoherence |
| Lasmiditan 50 mg vs.  Ubrogepant 25 mg | indirect | Very low | Serious Imprecisions, Heterogeneity |
| Lasmiditan 50 mg vs.  Ubrogepant 50 mg | indirect | Very low | Serious Imprecisions, Heterogeneity |
| Lasmiditan 50 mg vs.  Zavegepant 10 mg | indirect | Very low | Serious Imprecisions, Incoherence |
| Lasmiditan 50 mg vs.  Zavegepant 20 mg | indirect | Very low | Serious Imprecisions, Heterogeneity |
| Lasmiditan 50 mg vs.  Zavegepant 5 mg | indirect | Very low | Serious Imprecisions, Incoherence |
| Rimegepant 75 mg vs.  Ubrogepant 100 mg | indirect | Low | Heterogeneity, Incoherence |
| Rimegepant 75 mg vs.  Ubrogepant 25 mg | indirect | Very low | Serious Imprecisions, Incoherence |
| Rimegepant 75 mg vs.  Ubrogepant 50 mg | indirect | Very low | Serious Imprecisions, Heterogeneity, Incoherence |
| Rimegepant 75 mg vs.  Zavegepant 10 mg | indirect | Very low | Serious Imprecisions, Incoherence |
| Rimegepant 75 mg vs.  Zavegepant 20 mg | indirect | Very low | Serious Imprecisions, Heterogeneity |
| Rimegepant 75 mg vs.  Zavegepant 5 mg | indirect | Very low | Serious Imprecisions, Heterogeneity |
| Ubrogepant 100 mg vs.  Zavegepant 10 mg | indirect | Very low | Serious Imprecisions, Incoherence |
| Ubrogepant 100 mg vs.  Zavegepant 20 mg | indirect | Very low | Serious Imprecisions, Heterogeneity, Incoherence |
| Ubrogepant 100 mg vs.  Zavegepant 5 mg | indirect | Very low | Serious Imprecisions, Heterogeneity, Incoherence |
| Ubrogepant 25 mg vs.  Zavegepant 10 mg | indirect | Very low | Serious Imprecisions, Incoherence |
| Ubrogepant 25 mg vs.  Zavegepant 20 mg | indirect | Very low | Serious Imprecisions, Heterogeneity |
| Ubrogepant 25 mg vs.  Zavegepant 5 mg | indirect | Very low | Serious Imprecisions, Heterogeneity |
| Ubrogepant 50 mg vs.  Zavegepant 10 mg | indirect | Very low | Serious Imprecisions, Heterogeneity, Incoherence |
| Ubrogepant 50 mg vs.  Zavegepant 20 mg | indirect | Very low | Serious Imprecisions, Incoherence |
| Ubrogepant 50 mg vs.  Zavegepant 5 mg | indirect | Very low | Serious Imprecisions, Heterogeneity |

Comparison: names of the interventions compared. Nature of evidence: mixed=combination of direct and indirect evidence, indirect=only indirect evidence available. Confidence level: high, moderate, low, very low. Downgrading: CINeMA items responsible for downgrading. CINeMA: Confidence in Network Meta-analysis.

# Fig. S1: Risk of bias assessment for the included studies.


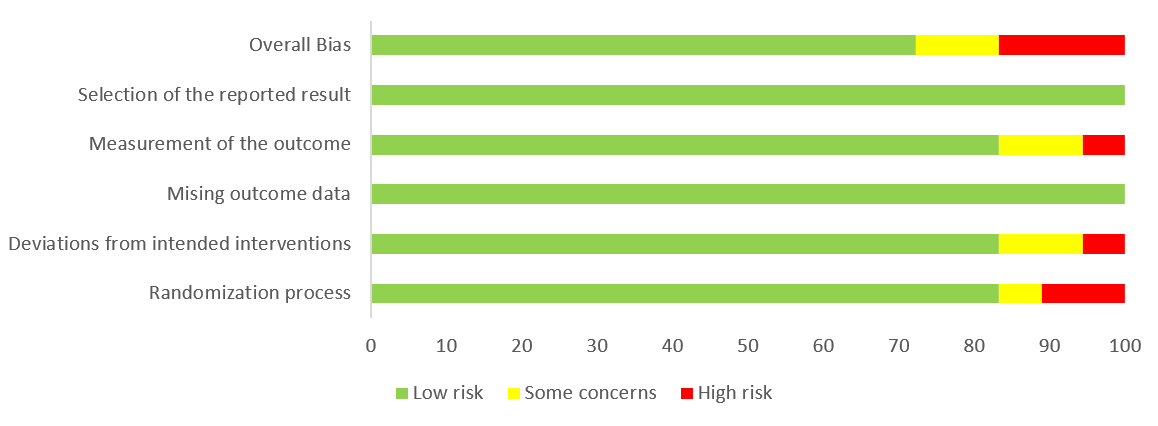

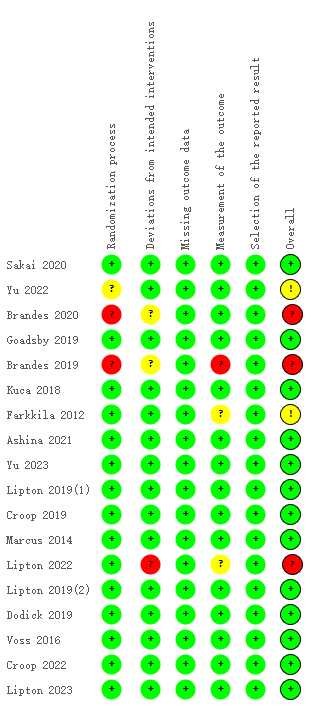


# Fig. S2: Network plot for pain freedom at 2 hours

The size of the nodes corresponds to the number of participants randomized to each treatment. Treatments with direct comparisons are linked with a line; its thickness corresponds to the number of trials evaluating the comparison.

# Fig. S3: Network plot for pain relief at 2 hours

The size of the nodes corresponds to the number of participants randomized to each treatment. Treatments with direct comparisons are linked with a line; its thickness corresponds to the number of trials evaluating the comparison

# Fig. S4: Network plot for MBS freedom at 2 hours

The size of the nodes corresponds to the number of participants randomized to each treatment. Treatments with direct comparisons are linked with a line; its thickness corresponds to the number of trials evaluating the comparison; MBS: most bothersome symptom.

# Fig. S5: Network plot for sustained pain freedom over 24 hours

The size of the nodes corresponds to the number of participants randomized to each treatment. Treatments with direct comparisons are linked with a line; its thickness corresponds to the number of trials evaluating the comparison.

# Fig. S6: Network plot for sustained pain relief over 24 hours

The size of the nodes corresponds to the number of participants randomized to each treatment. Treatments with direct comparisons are linked with a line; its thickness corresponds to the number of trials evaluating the comparison.

# Fig. S7: Network plot for freedom from photophobia at 2 hours

The size of the nodes corresponds to the number of participants randomized to each treatment. Treatments with direct comparisons are linked with a line; its thickness corresponds to the number of trials evaluating the comparison.

# Fig. S8: Network plot for freedom from phonophobia at 2 hours

The size of the nodes corresponds to the number of participants randomized to each treatment. Treatments with direct comparisons are linked with a line; its thickness corresponds to the number of trials evaluating the comparison.

# Fig. S9: Confidence in evidence for all drugs compared to placebo


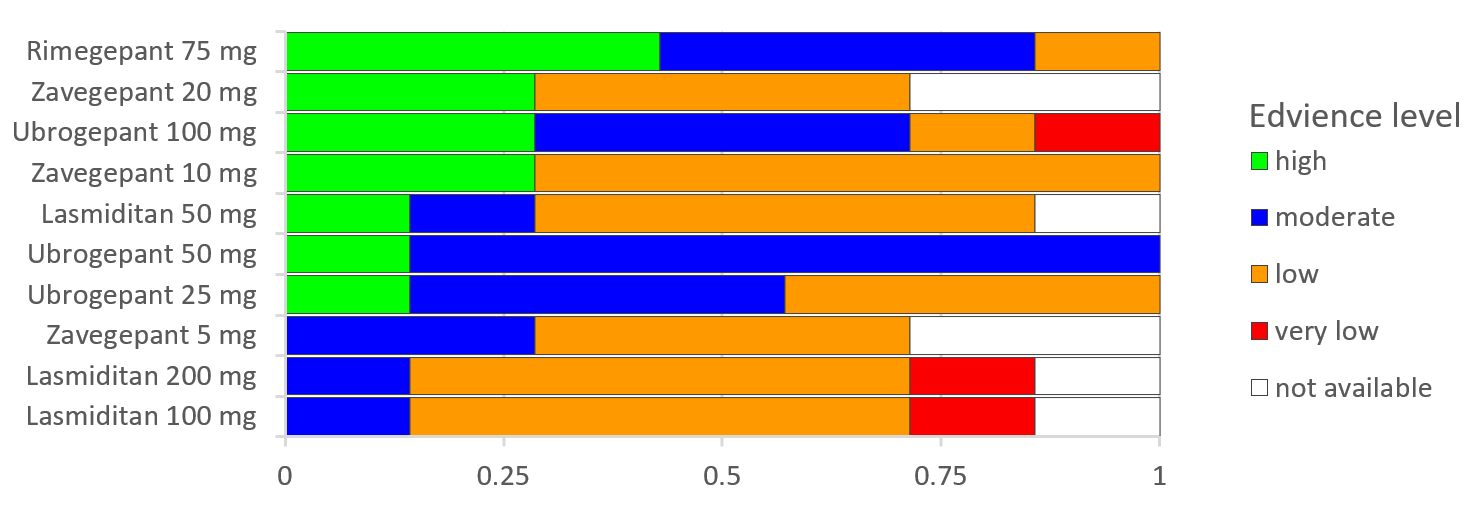


Confidence in evidence for all drugs used to treat acute migraine compared to placebo in this figure we present a summary of the confidence in the evidence for the individual drugs compared to placebo according to CINeMA (Confidence in Network Meta-analysis). Seven outcomes were considered. The bars present the percentage of outcomes with each evidence level (e.g. for ubrogepant 100 mg 29% of the reported outcomes had a high evidence level, 43% a moderate, 14% a low and 14% a very low one). The drugs with the largest proportion of outcomes ranked with high certainty of evidence are presented on top (e.g. for Rimegepant 75 mg 43% of the outcomes had a high level of evidence). As CINeMA does not consider comparisons for which no data are available, we added this information in the white bars (e.g. for Zavegepant 20 mg for 29% of the outcomes no data were available at all). Colour code: green=high, blue=moderate, orange=low, red=very low, white=percentage of outcomes with no data available.

# Reference list of included trials

1. Sakai F, Takeshima T, Homma G, Tanji Y, Katagiri H, Komori M. Phase 2 randomized placebo-controlled study of lasmiditan for the acute treatment of migraine in Japanese patients. *Headache*. May 2021;61(5):755-765. doi:10.1111/head.14122

2. Yu T, He L, Yang X, et al. Efficacy and Safety of Lasmiditan as a Novel Acute Treatment in Chinese Patients with Migraine: A Subpopulation Analysis of the Randomized Controlled Phase 3 CENTURION Trial. *Neurol Ther*. Sep 2022;11(3):1269-1283. doi:10.1007/s40120-022-00369-1

3. Brandes JL, Klise S, Krege JH, et al. Long-term safety and efficacy of lasmiditan for acute treatment of migraine: final results of the GLADIATOR study. Journal article. *Cephalalgia reports*. 2020;3doi:10.1177/2515816320958176

4. Goadsby PJ, Wietecha LA, Dennehy EB, et al. Phase 3 randomized, placebo-controlled, double-blind study of lasmiditan for acute treatment of migraine. *Brain*. Jul 1 2019;142(7):1894-1904. doi:10.1093/brain/awz134

5. Brandes JL, Klise S, Krege JH, et al. Interim results of a prospective, randomized, open-label, Phase 3 study of the long-term safety and efficacy of lasmiditan for acute treatment of migraine (the GLADIATOR study). *Cephalalgia*. Oct 2019;39(11):1343-1357. doi:10.1177/0333102419864132

6. Kuca B, Silberstein SD, Wietecha L, et al. Lasmiditan is an effective acute treatment for migraine A phase 3 randomized study. Article. *Neurology*. Dec 11 2018;91(24):E2222-E2232. doi:10.1212/wnl.0000000000006641

7. Farkkila M, Diener H-C, Geraud G, et al. Efficacy and tolerability of lasmiditan, an oral 5-HT1F receptor agonist, for the acute treatment of migraine: a phase 2 randomised, placebo-controlled, parallel-group, dose-ranging study. Article. *Lancet Neurology*. May 2012;11(5):405-413. doi:10.1016/s1474-4422(12)70047-9

8. Ashina M, Reuter U, Smith T, et al. Randomized, controlled trial of lasmiditan over four migraine attacks: Findings from the CENTURION study. *Cephalalgia*. Mar 2021;41(3):294-304. doi:10.1177/0333102421989232

9. Yu S, Kim BK, Guo A, et al. Safety and efficacy of rimegepant orally disintegrating tablet for the acute treatment of migraine in China and South Korea: a phase 3, double-blind, randomised, placebo-controlled trial. Journal article. *The lancet Neurology*. 2023;22(6):476‐484. doi:10.1016/S1474-4422(23)00126-6

10. Lipton RB, Croop R, Stock EG, et al. Rimegepant, an Oral Calcitonin Gene-Related Peptide Receptor Antagonist, for Migraine. Article. *New England Journal of Medicine*. Jul 11 2019;381(2):142-149. doi:10.1056/NEJMoa1811090

11. Croop R, Goadsby PJ, Stock DA, et al. Efficacy, safety, and tolerability of rimegepant orally disintegrating tablet for the acute treatment of migraine: a randomised, phase 3, double-blind, placebo-controlled trial. Article. *Lancet*. Aug 31 2019;394(10200):737-745. doi:10.1016/s0140-6736(19)31606-x

12. Marcus R, Goadsby PJ, Dodick D, Stock D, Manos G, Fischer TZ. BMS-927711 for the acute treatment of migraine: A double-blind, randomized, placebo controlled, dose-ranging trial. Article. *Cephalalgia*. Feb 2014;34(2):114-125. doi:10.1177/0333102413500727

13. Lipton RB, Dodick DW, Goadsby PJ, et al. Efficacy of Ubrogepant in the Acute Treatment of Migraine With Mild Pain vs Moderate or Severe Pain. *Neurology*. Oct 25 2022;99(17):e1905-e1915. doi:10.1212/wnl.0000000000201031

14. Lipton RB, Dodick DW, Ailani J, et al. Effect of Ubrogepant vs Placebo on Pain and the Most Bothersome Associated Symptom in the Acute Treatment of Migraine: The ACHIEVE II Randomized Clinical Trial. *Jama*. Nov 19 2019;322(19):1887-1898. doi:10.1001/jama.2019.16711

15. Dodick DW, Lipton RB, Ailani J, et al. Ubrogepant for the Treatment of Migraine. Journal article. *New England journal of medicine*. 2019;381(23):2230‐2241. doi:10.1056/NEJMoa1813049

16. Voss T, Lipton RB, Dodick DW, et al. A phase IIb randomized, double-blind, placebo-controlled trial of ubrogepant for the acute treatment of migraine. Article. *Cephalalgia*. Aug 2016;36(9):887-898. doi:10.1177/0333102416653233

17. Croop R, Madonia J, Stock DA, et al. Zavegepant nasal spray for the acute treatment of migraine: A Phase 2/3 double-blind, randomized, placebo-controlled, dose-ranging trial. *Headache*. Oct 2022;62(9):1153-1163. doi:10.1111/head.14389

18. Lipton RB, Croop R, Stock DA, et al. Safety, tolerability, and efficacy of zavegepant 10 mg nasal spray for the acute treatment of migraine in the USA: a phase 3, double-blind, randomised, placebo-controlled multicentre trial. Randomized Controlled Trial; Multicenter Study; Clinical Trial, Phase III; ; Research Support, Non-U.S. Gov't. *The Lancet Neurology*. 2023-03 2023;22(3):209-217. doi:10.1016/s1474-4422(22)00517-8
